# Supplementary material for: Tyrosine phosphorylation of the GARU E3 ubiquitin ligase promotes gibberellin signalling by preventing GID1 degradation
Source: Nat Commun. 2017 Oct 17;8:1004. doi: 10.1038/s41467-017-01005-5 (PMC5645313; doi:10.1038/s41467-017-01005-5)
Supplement: Supplementary file 1 — Supplementary Information [file 41467_2017_1005_MOESM1_ESM.pdf]

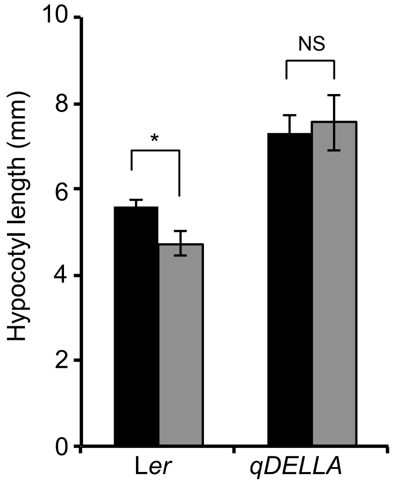

Supplementary Figure 1: GNS-induced inhibition of hypocotyl elongation in Ler and *qDELLA* mutant.

L*er* and *qDELLA* mutant (*gai-t6 rga-t2 rgl1-1 rgl3-1 rgl2-1/SGT625-5* quintuple *della* mutant, CS16298 form ABRC) seedlings were transferred on half-strength MS agar plates (1% sucrose) supplemented with (grey bars) or without (black bars) of 10 μM GNS. After the plates were wrapped in aluminum foil, and incubated in a growth cabinet for 5 days. Hypocotyl length was measured using ImageJ software. Data are means (± s.d.) of three independent experiments, n=21. A student's t-test was calculated at the probability of either 5% (*$P < 0.05$). NS; not significant

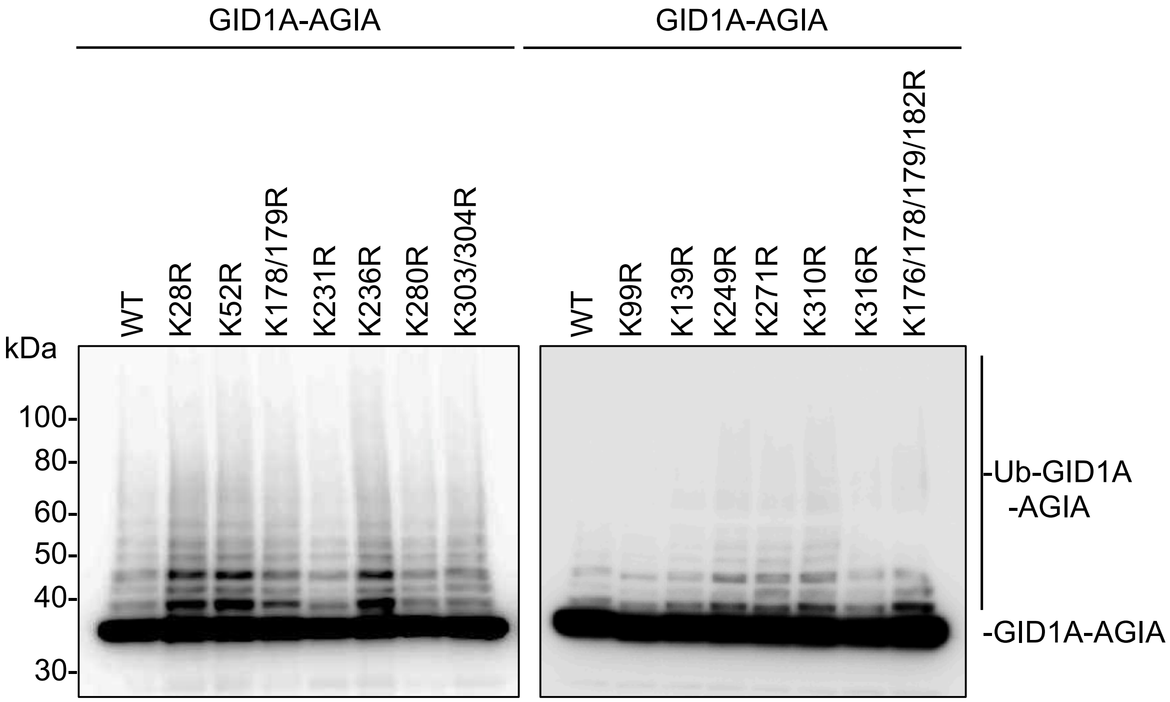

Supplementary Figure 2: In vitro ubiquitination assay.
GID1A-AGIA and its mutants (K28R, K52R, K178/179R, K231R, K236R, K280R, K303/304R, K99R, K139R, K249R, K271R, K310R, K316R, and K176/178/179/182R) were incubated with GARU and recombinant E2. GID1A-AGIA proteins were detected by immunoblot analysis using anti-AGIA antibody.

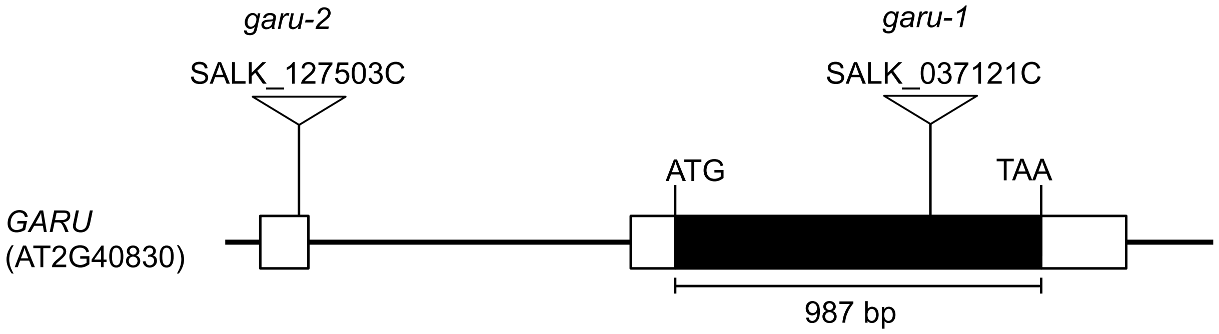

Supplementary Figure 3: Position of T-DNA insertion in *GARU* gene.
Positions of T-DNA insertions in *GARU* gene. Exons, introns and 5'/3' UTR are represented by black boxes, lines and white boxes, respectively.

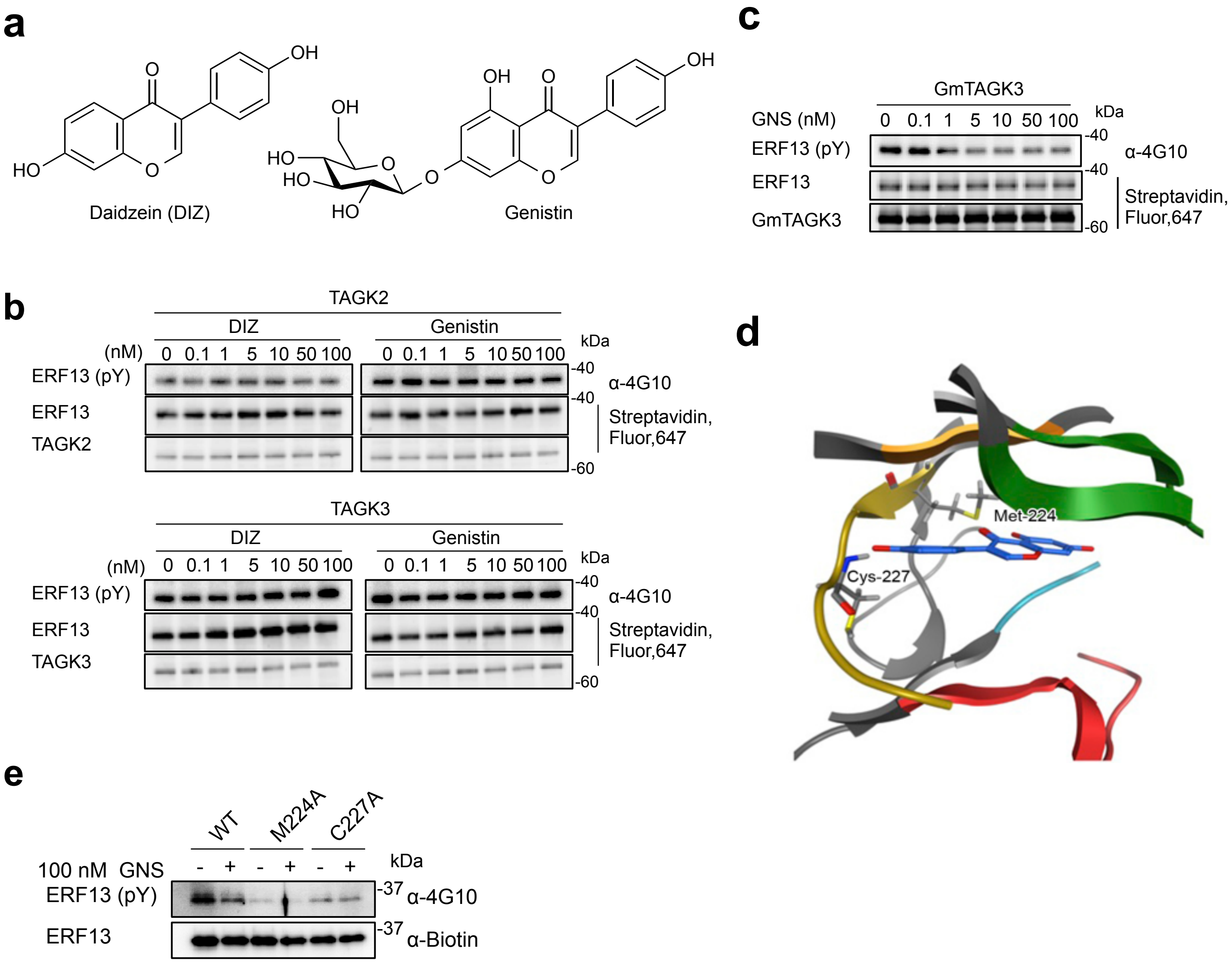

Supplementary Figure 4: TAGK activities are inhibited by GNS.
(**a**) Structures of GNS analogs. (**b**) In vitro inhibition assay of TAGKs by daidzein (DIZ) and genistin on TAGK2 and TAGK3 activities. Tyr-phosphorylation was detected by anti-phospho-Tyr antibody (4G10). Biotin-labeled proteins were detected using streptavidin Alexa Fluor, 647. ERF13 is substrate for TAGK2 and TAGK3. (**c**) In vitro inhibition assay of GmTAGK3 using GNS. (**d**) Generated models of the AtTAGK3 kinase domain interacting with GNS. Close-up views of a representative binding model for genistein in the ATP-binding pocket of AtTAGK3 kinase domain are shown. GNS is colored by blue. Regions including Gly-rich loop, HRD motif, DGF motif, hinge region and the invariant Lys interacting with ß-phosphoryl groups of the ATP are colored by green, red, light blue, yellow and orange, respectively (Supplementary Note, Supplementary Fig. 5). (**e**) In vitro kinase assay using two mutants of the associate amino acids predicted from TAGK3 structural modeling. (pY) ;Tyr-phosphorylation (c, d, e).

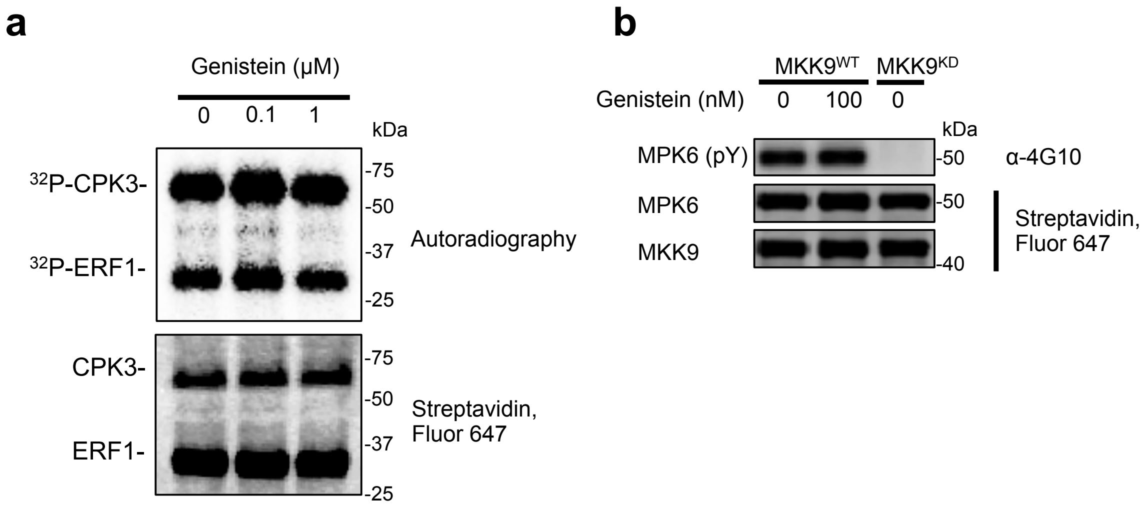

Supplementary Figure 5: In vitro inhibition assay of CPK3 and MKK9 by genistein.
(**a**) Biotinylated-CPK3 and its substrate, biotinylated-ERF1, were incubated in the kinase buffer supplement with 37 kBq of [γ-32P] ATP, 100 μM $CaCl_2$ and genistein (0.1 μM or 1 μM). Phosphorylation signal was detected by autoradiography (upper panel), and protein level was detected by immunoblot analysis using Streptavidin, Fluor 647 (under panel). (**b**) After the incubation of biotinylated-MKK9 with biotinylated substrate MPK6, in the presence of 100 nM genistein or 1% DMSO (control), Tyr phosphorylation was detected by immunoblot analysis with anti-pTyr antibody (4G10) (Upper panel). Biotinylated proteins were detected by Streptavidin, Fluor 647 (substrate protein; middle panel, MKK9; under panel). WT; wild-type, KD; kinase-dead form.

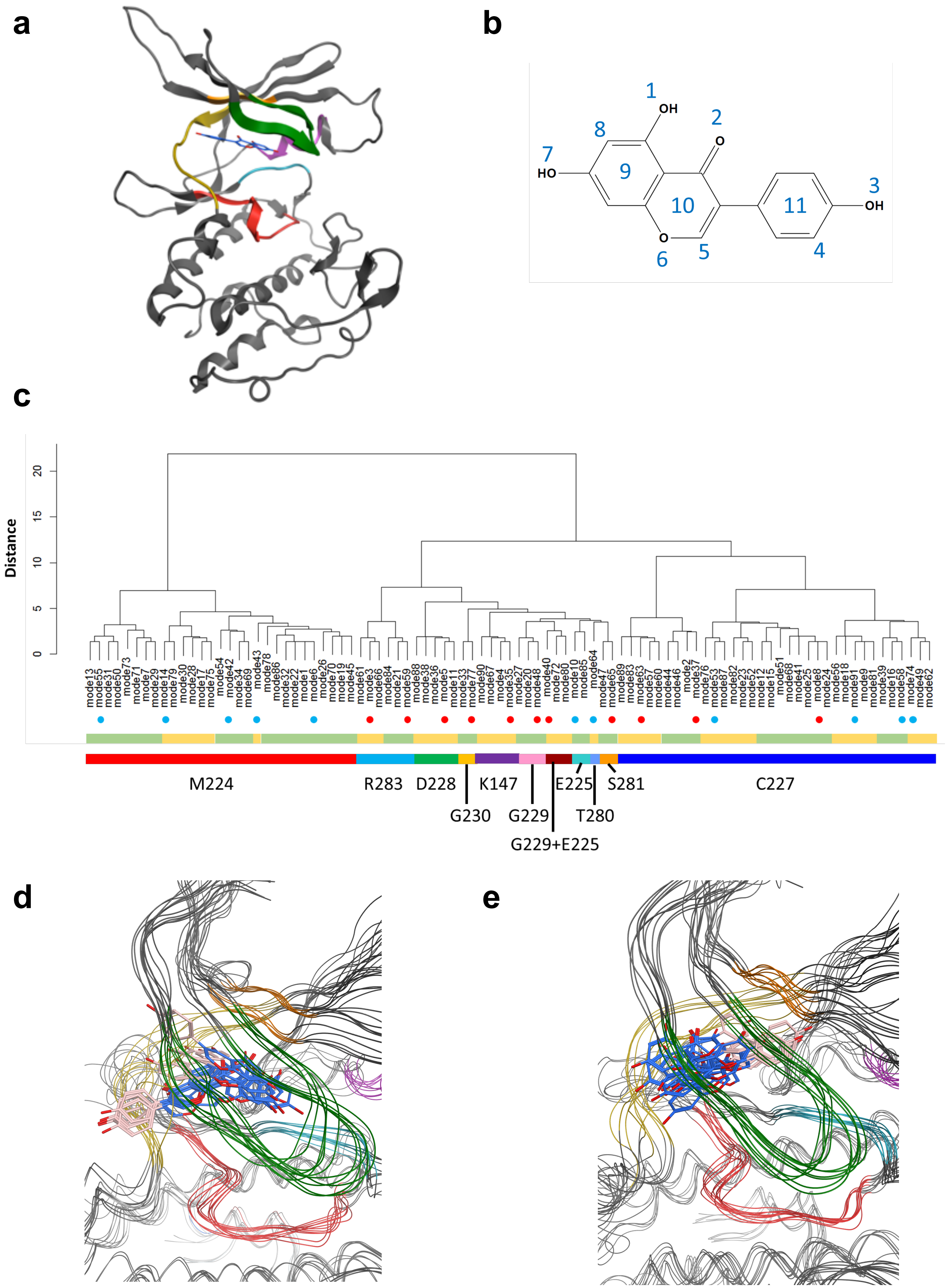

a
b
1
OH
2
O
8
7
HO
9
10
11
3
OH
O
6
5
4
c
Distance
0
5
10
15
20
M224
R283
D228
K147
E225
S281
C227
G230
G229
T280
G229+E225
d
e

Supplementary Figure 6: Binding Poses of the AtTAGK3 Kinase Domain Interacting with Genistein

(**a**) Global view of a potential binding pose (mode 6 in panel c) for genistein (GNS) in the ATP-binding pocket of the AtTAGK3 kinase domain. GNS is colored as blue. Regions including Gly rich loop, HRD motif, DGF motif, hinge region, αC-helix, and the invariant Lys interacting with β-phosphoryl groups of ATP are colored respectively as green, red, light blue, yellow, purple, and orange. (**b**) Positions of GNS interacting with the ATP-binding pocket. The numbers for positions are assigned for the sake of argument. (**c**) Hierarchical clustering dendrogram of the 91 binding poses. The vertical scale shows the inter-cluster distance based on the fingerprints. The lowest free-energy pose in each cluster is indicated by the light blue or red circle which represents the original type of inhibitor orientation in the ATP-binding pocket of nine kinase domains. The poses were derived from templates with inhibitors whose benzopyrone structures were located respectively inside (light blue) and outside (red) of the ATP-binding pocket. Upper and lower color bars show the 22 representative clusters and the classification of the predicted poses based on residues that interact with the OH group at the 5-site of the two aromatic rings of GNS (position 1 in panel b) in the ATP-binding pocket of the AtTAGK3 kinase domain. The selected poses, indicated by the light blue and red circles in panel c, whose benzopyrone structures are located inside (**d**) and outside (**e**) of the ATP-binding pocket are superimposed, respectively. The benzopyrone structure is shown as blue stick model in GNS.

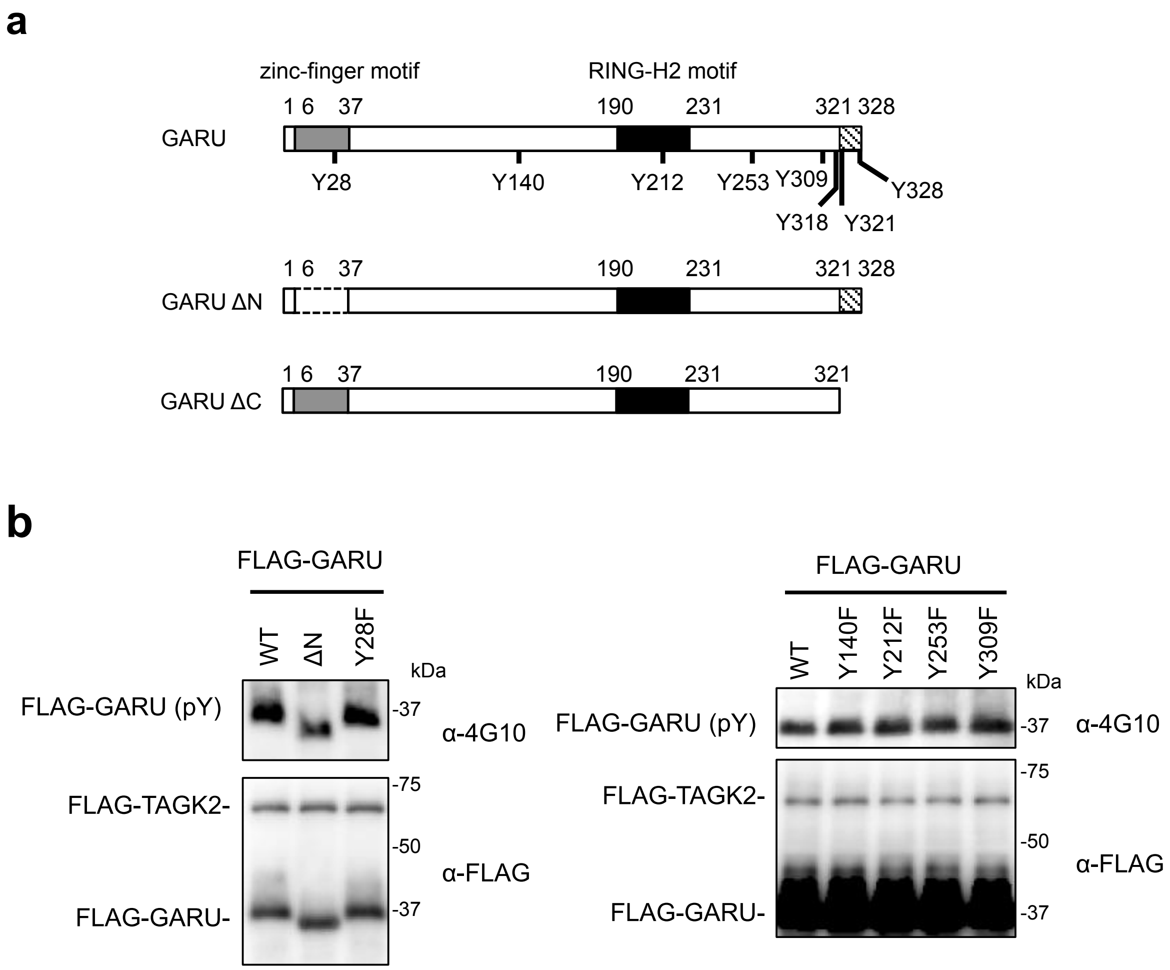

Supplementary Figure 7: In vitro phosphorylation analysis of GARU mutants.
(**a**) Diagram of the structure of full-length GARU, GARU ΔN and GARU ΔC mutants. (**b**) *In vitro* kinase assay of wild-type GARU and its mutants. Five Tyr residues (Y28, Y140, Y212, Y253 and Y309) were changed to Phe residue in GARU. Wild-type GARU, GARU ΔN and five Phe mutants were used for *in vitro* kinase assay with TAGK2. Tyr phosphorylation was detected by immunoblot analysis with anti-pTyr antibody (4G10). FLAG-GARU and FLAG-TAGK2 proteins were detected by anti-FLAG. (pY); Tyr-phosphorylation.

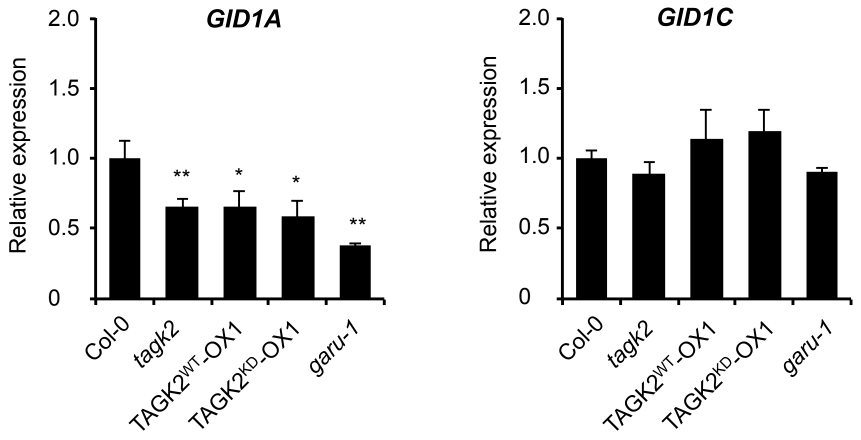

Supplementary Figure 8: RT-qPCR analysis of GID1A and GID1C genes in seedling of Col-0*, tagk2,* TAGK2^{WT}-OX1, TAGK2^{KD}-OX1*, and garu-1.*

Total RNA was isolated form 5-days-old seedling, and transcript levels were analyzed by RT-qPCR. The transcript levels were normalized to the level of ACTIN2, and relative transcript level Col-0 as one. Experiment had three biological repeats and the average value is shown with s.e.m. (n=9). Statistically significant changes compared Col-0 are indicated (**P<0.01, *P < 0.05, as determined using student's t-test).

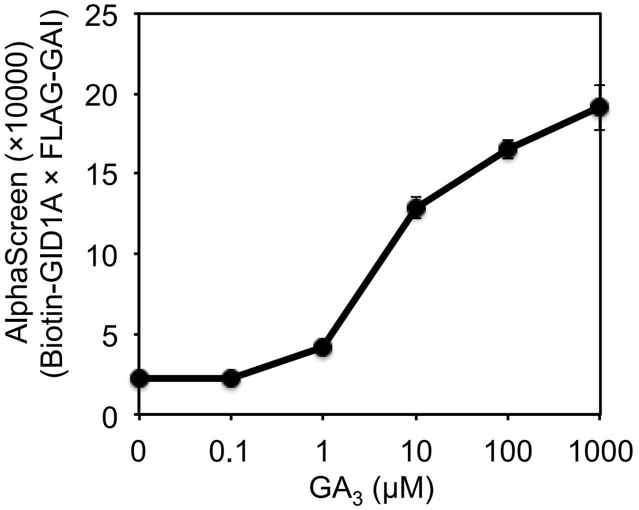

Supplementary Figure 9: Protein-protein interaction analysis of GID1A and GAI by AlphaScreen.

Biotinylated GID1A (Biotin-GID1A) and FLAG-GAI were incubated in the presence of GA_3. Interaction between GID1A and GAI was analyzed by AlphaScreen tecnology. Data are means (± s.d.) of three independent experiments.

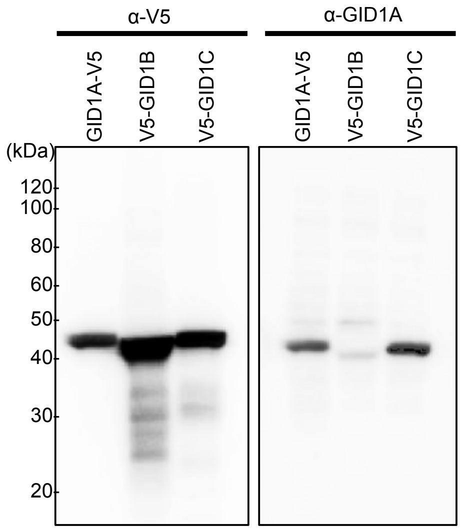

Supplementary Figure 10: Specificity analysis of anti-GID1A antibody. Specificity of anti-GID1A antibody was tested using recombinant V5-tagged GID1A, GID1B and GID1C proteins. All three GID1 proteins were analysed anti-V5 (left) and anti-GID1A antibody (right)

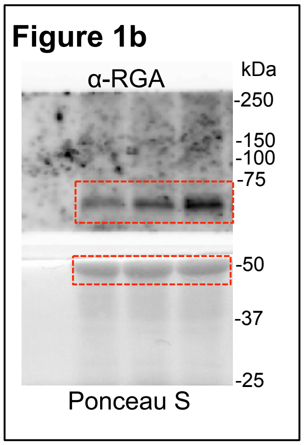

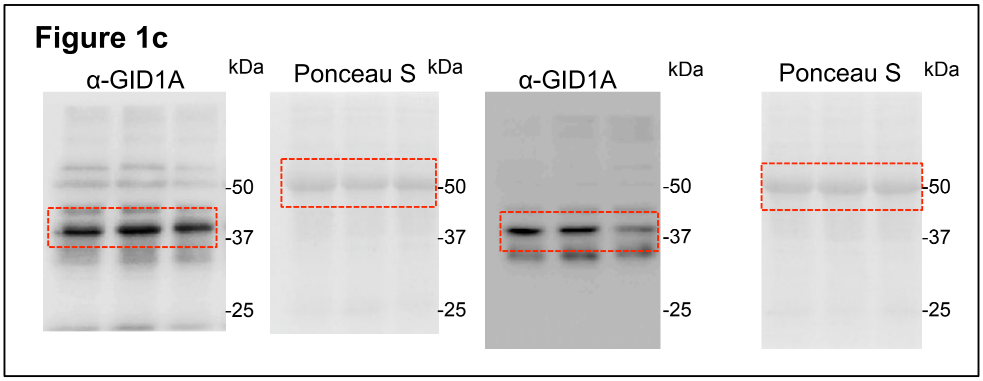

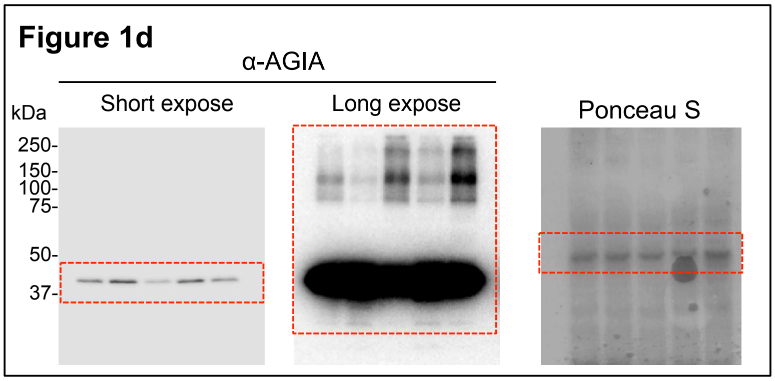

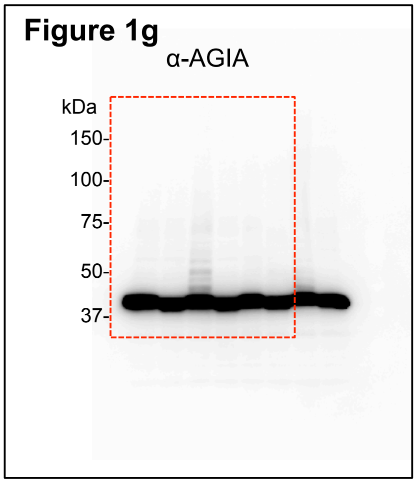

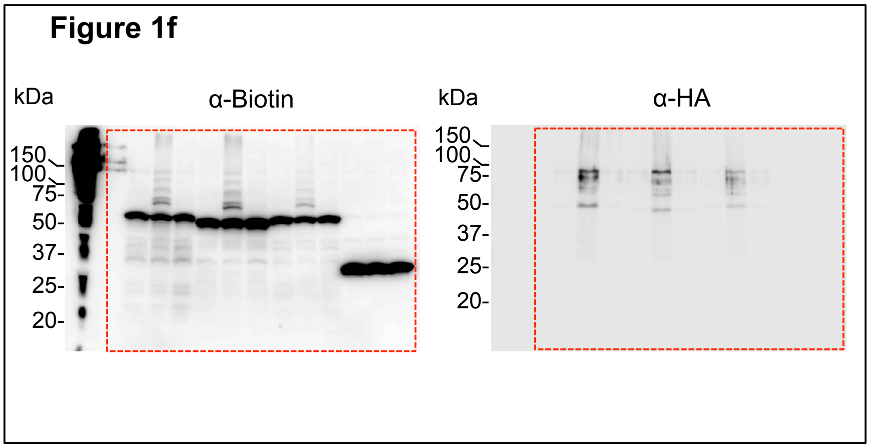

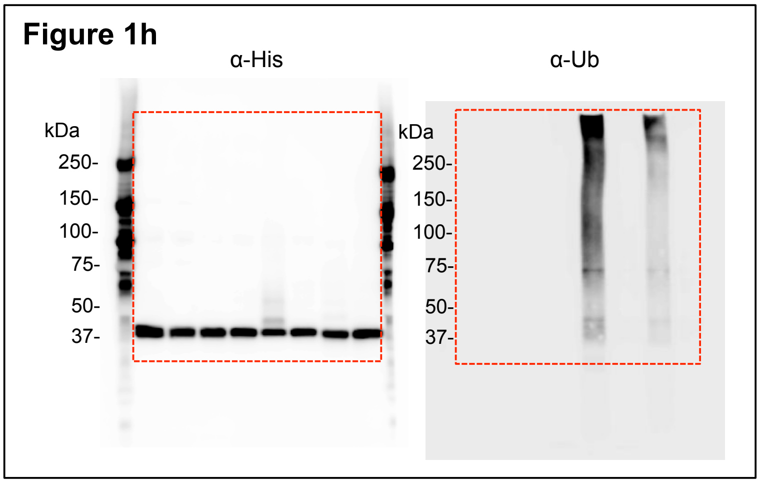

Supplementary Figure 11: Uncropped blot images.

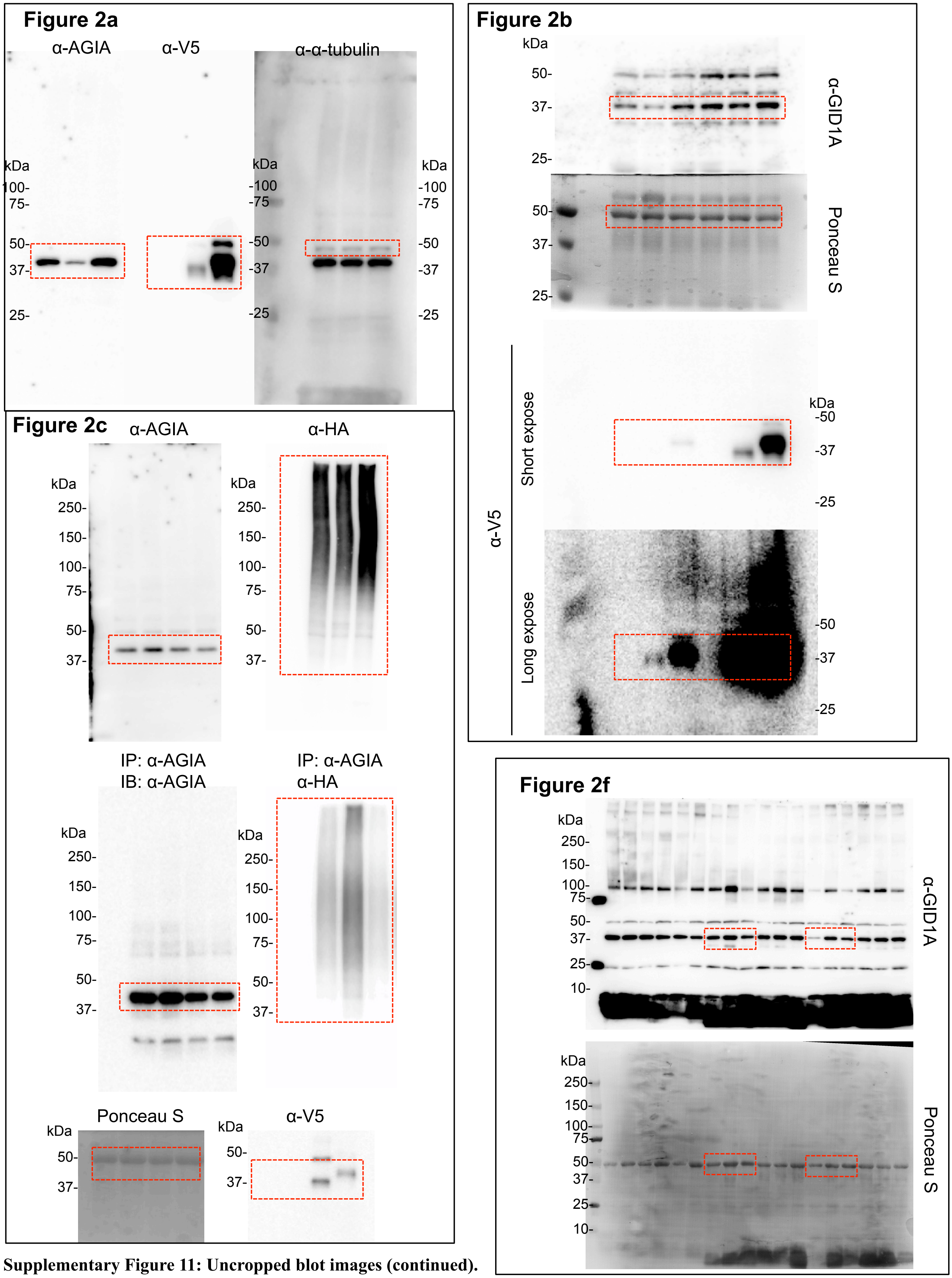

Supplementary Figure 11: Uncropped blot images (continued).

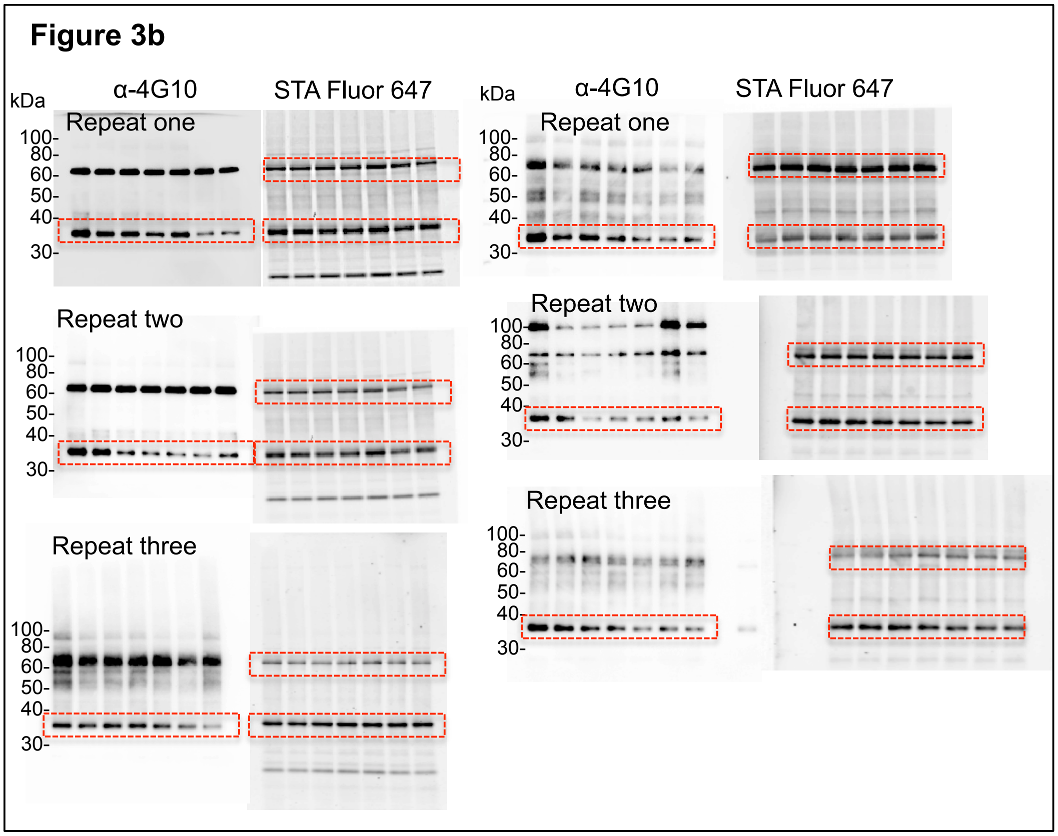

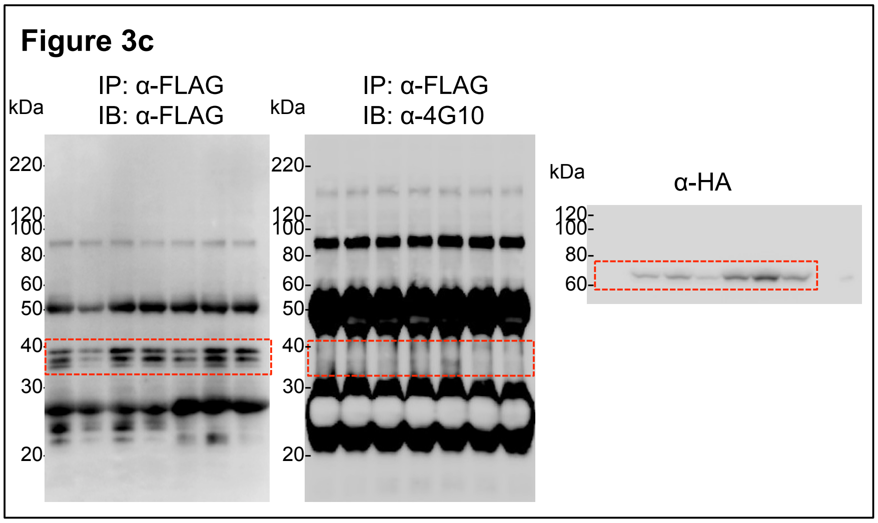

Supplementary Figure 11: Uncropped blot images (continued).

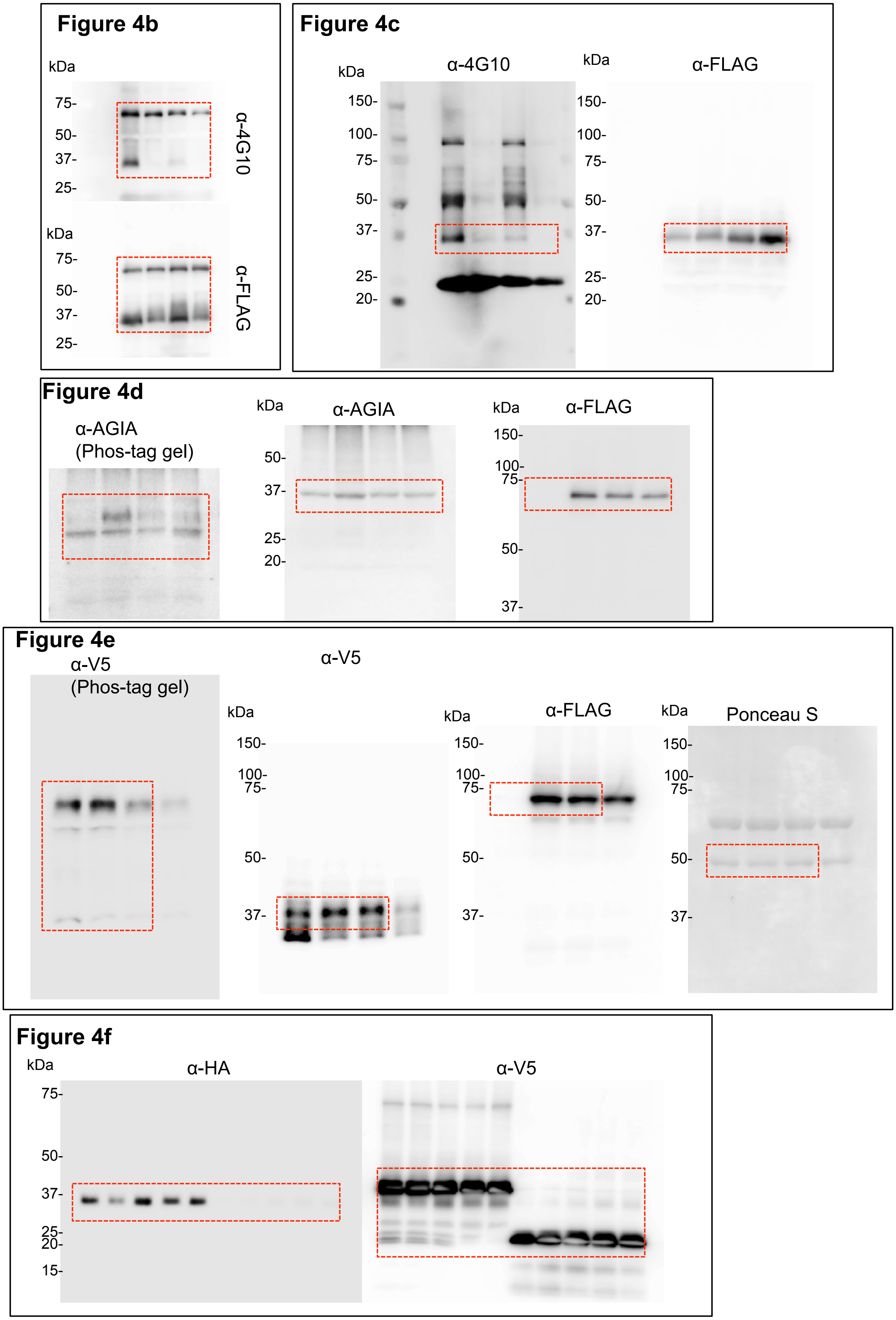

Supplementary Figure 11: Uncropped blot images (continued).

Figure 4g

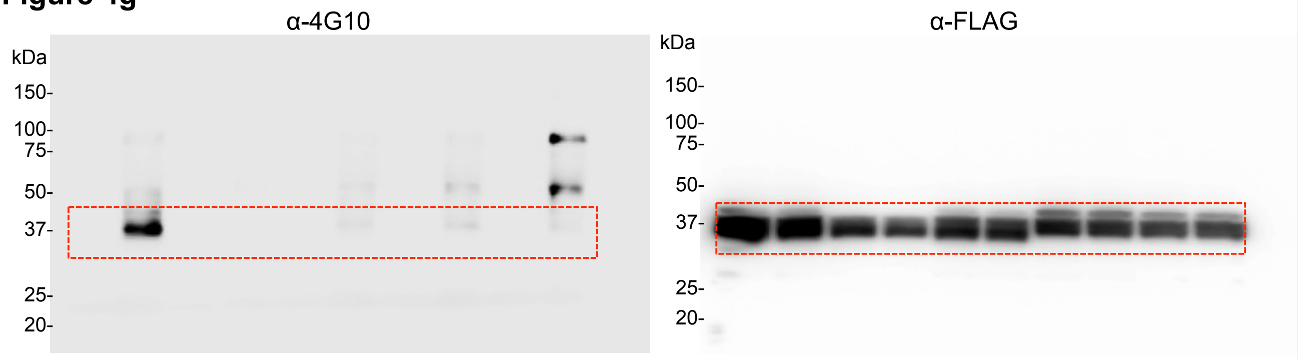

Figure 4h

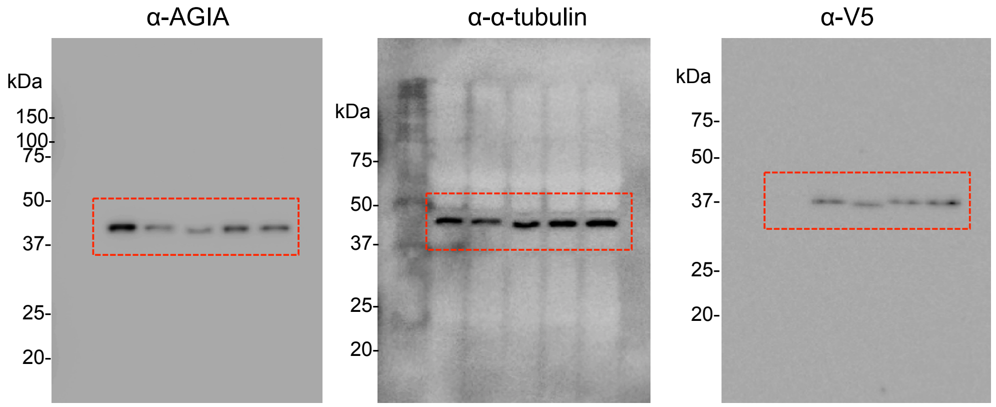

Figure 4i

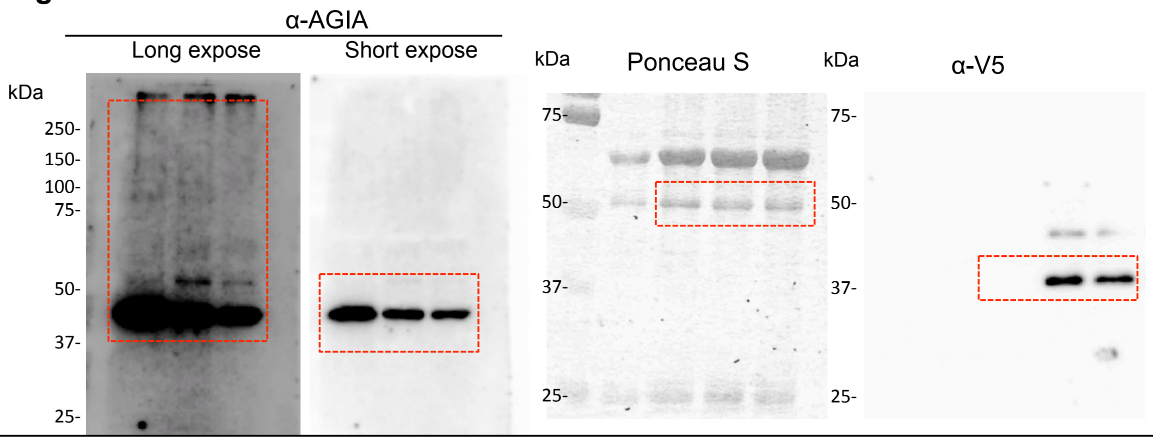

Supplementary Figure 11: Uncropped blot images (continued).

Figure 4j

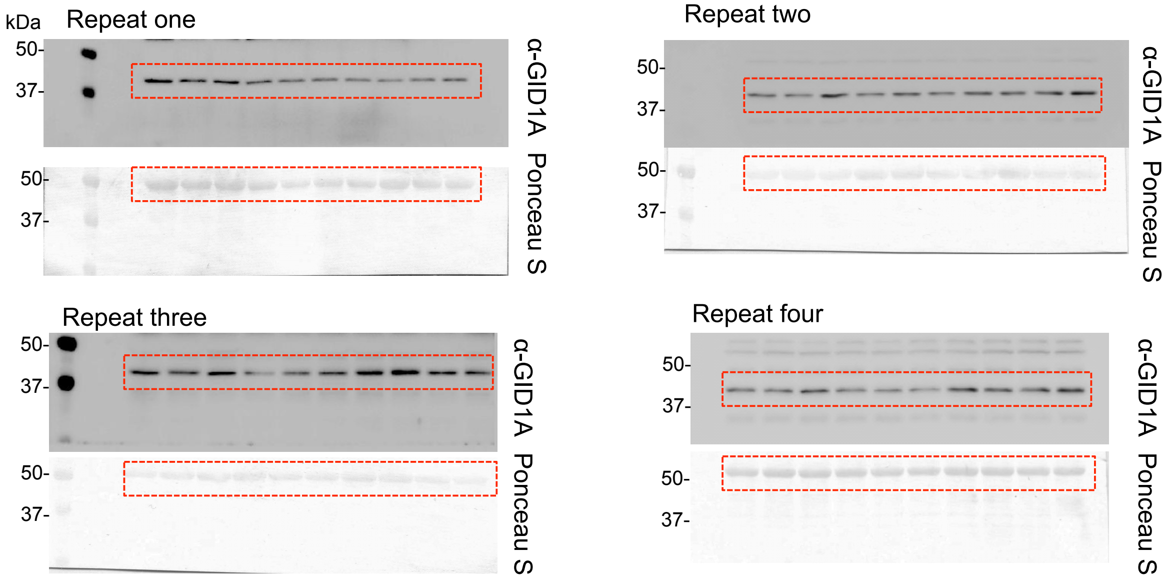

Figure 5b

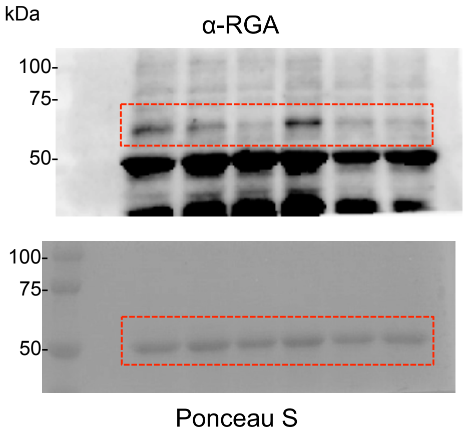

Figure 5e

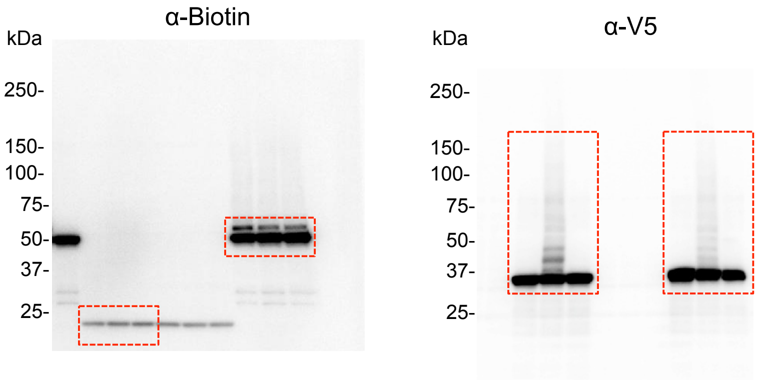

Figure 5f

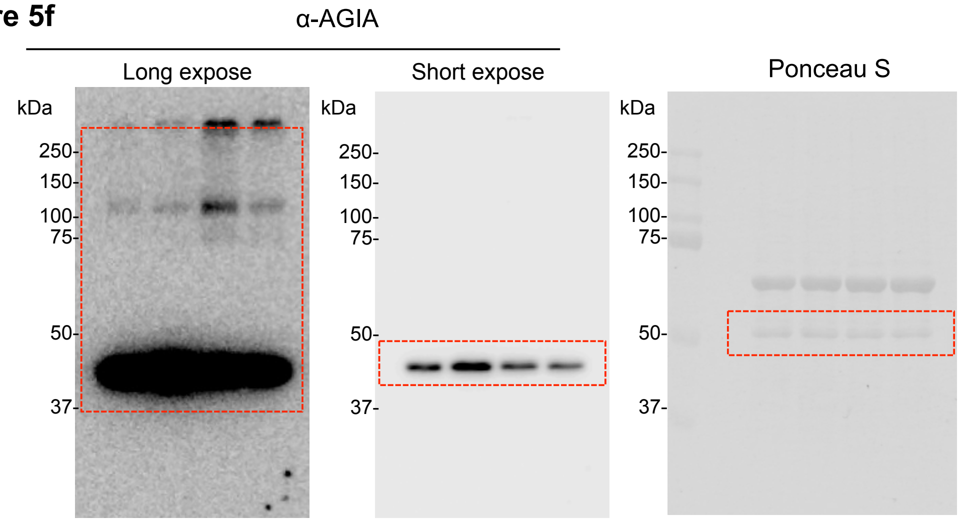

Supplementary Figure 11: Uncropped blot images (continued).

Figure 6a

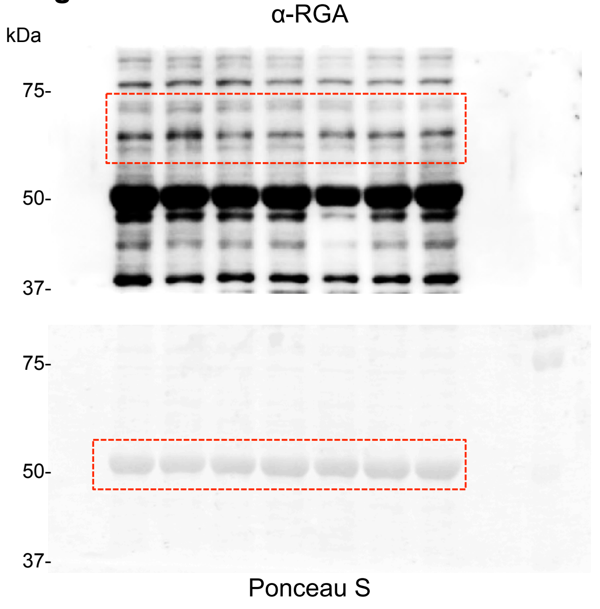

Figure 7a

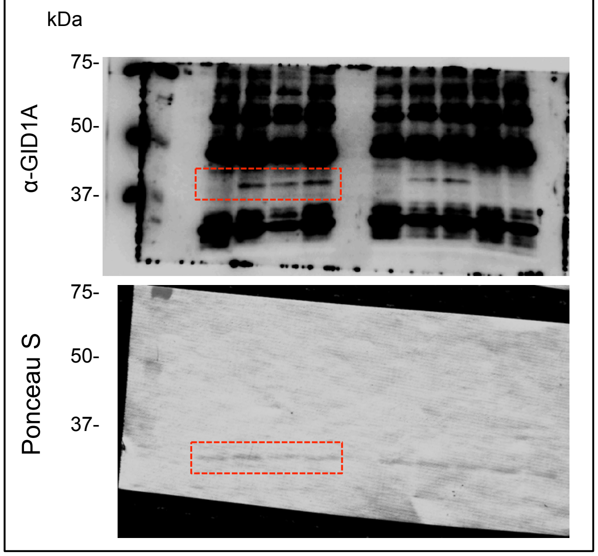

Figure 6b

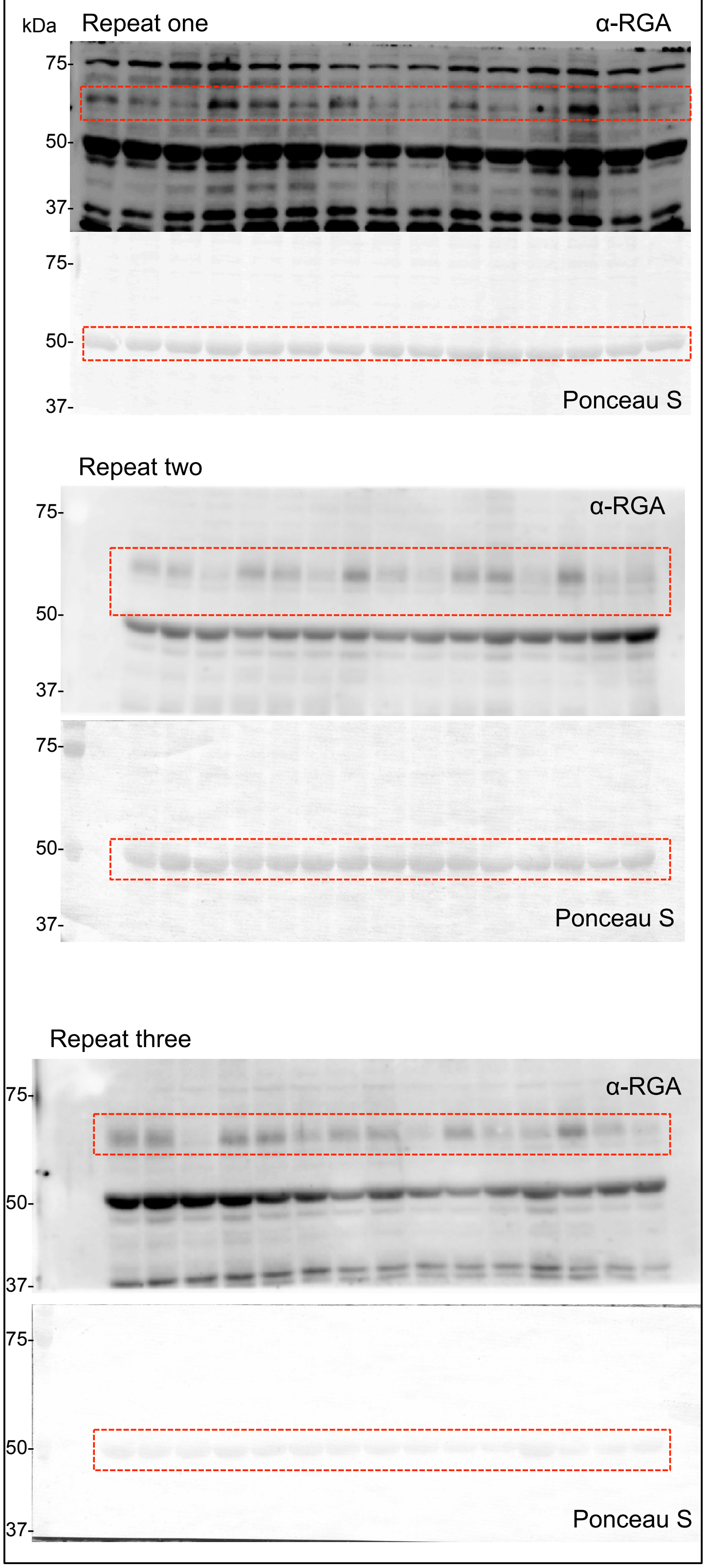

Supplementary Figure 11: Uncropped blot images (continued).

Figure 7c

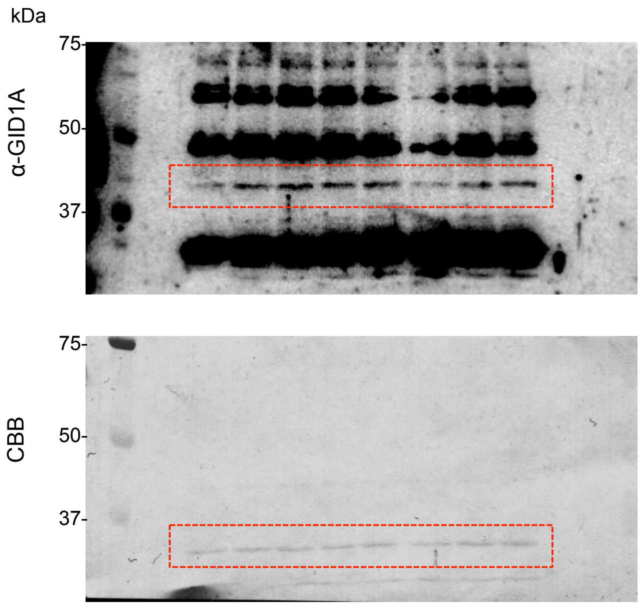

Figure 7d, e

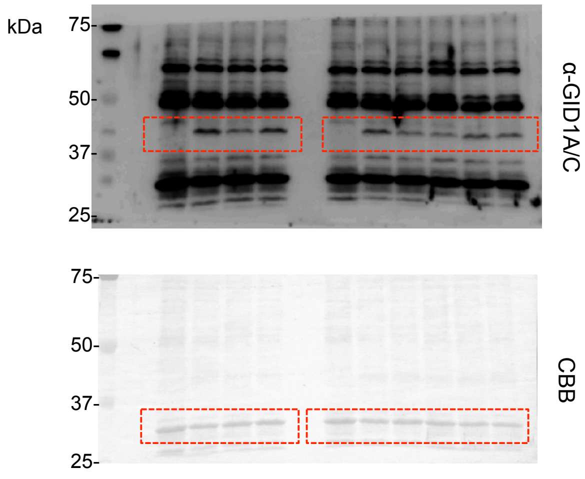

Figure 7f

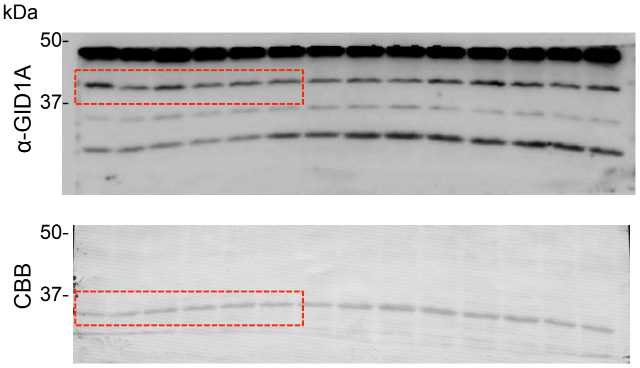

Supplementary Figure 2

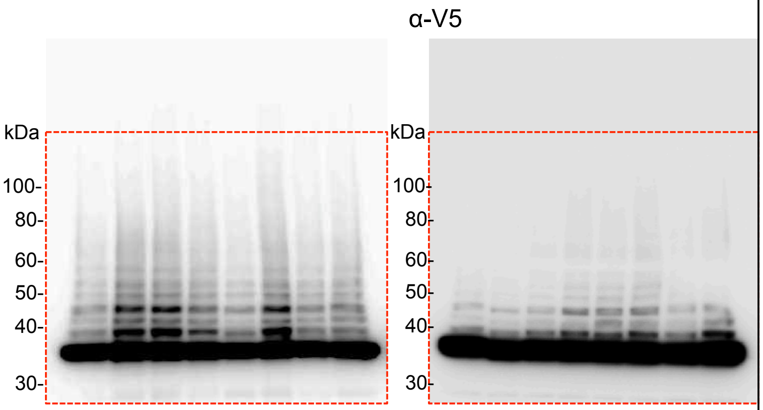

Supplementary Figure 4c

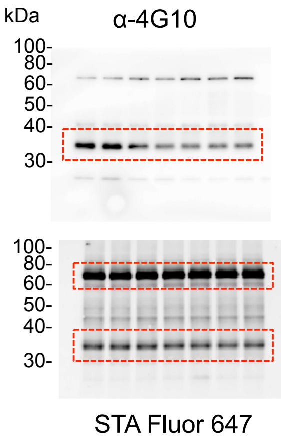

Supplementary Figure 11: Uncropped blot images (continued).

Supplementary 4d

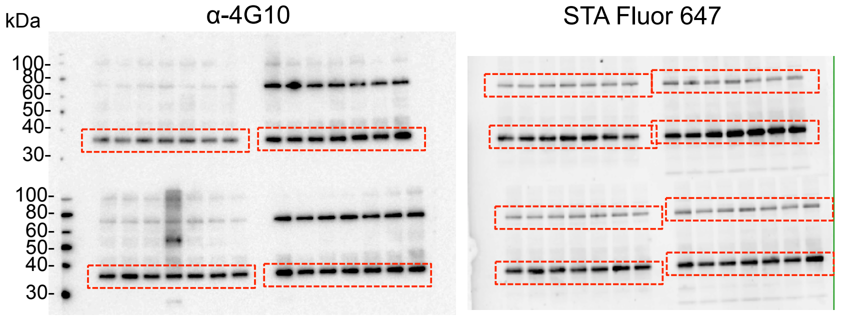

Supplementary 4e

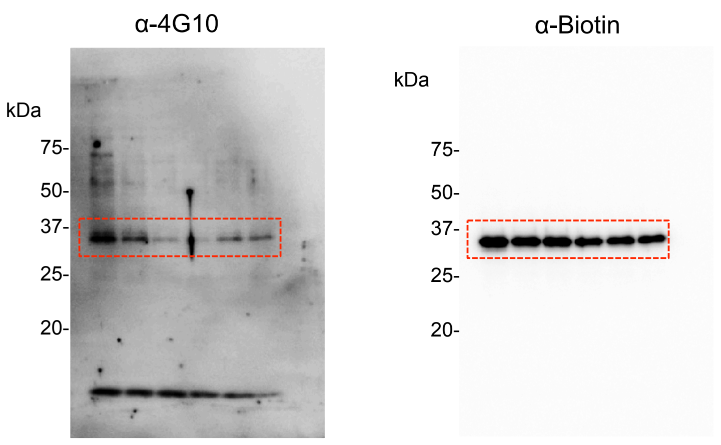

Supplementary Figure 5b

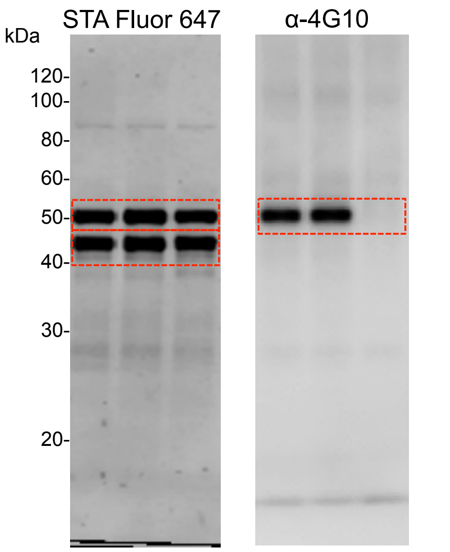

Supplementary Figure 5a

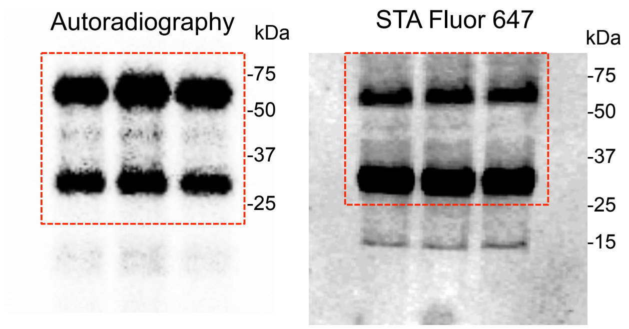

Supplementary Figure 11: Uncropped blot images (continued).

Supplementary Figure 7b

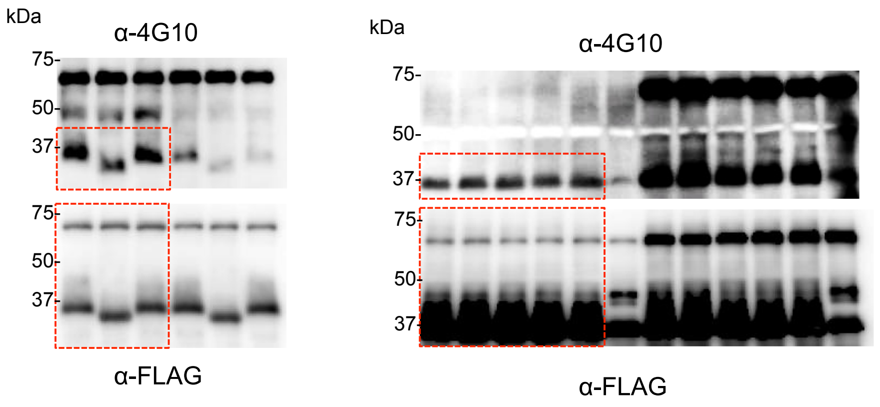

Supplementary Figure 10

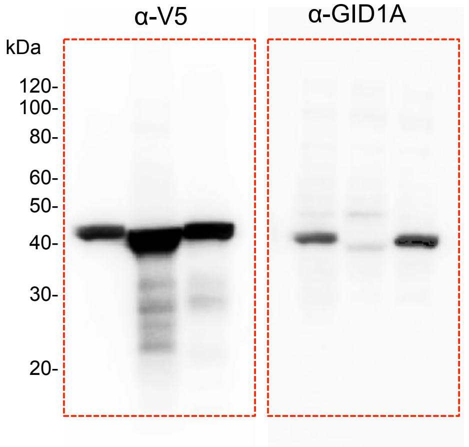

Supplementary Figure 11: Uncropped blot images (continued).

Supplementary Table 1: Interaction analysis of biotinylated GID1A with FLAG tagged RING E3 uqiquitin ligases using AlphaScreen.

Number	ID No.	Protein code	Function	Relative AlphaScreen signal (GID1A/DHFR)
1	AtE301A01	at1g45976	expressed protein	1.27
2	AtE301A02	at1g03770	zinc finger (C3HC4type RING finger) family protein	2.05
3	AtE301A03	at5g17600	zinc finger (C3HC4type RING finger) family protein	1.07
4	AtE301A04	at1g22510	zinc finger (C3HC4type RING finger) family protein	0.96
5	AtE301A05	at1g50410	SNF2 domaincontaining protein / helicase domaincontaining protein / RING finger domaincontaining protein	0.92
6	AtE301A06	at1g63900	zinc finger (C3HC4type RING finger) family protein	3.72
7	AtE301A07	at1g22500	zinc finger (C3HC4type RING finger) family protein	3.07
8	AtE301A08	at1g49200	zinc finger (C3HC4type RING finger) family protein	3.86
9	AtE301A09	at3g15070	zinc finger (C3HC4type RING finger) family protein	1.74
10	AtE301A11	at3g09760	zinc finger (C3HC4type RING finger) family protein	4.31
11	AtE301A12	at1g68820	membrane protein, putative	1.33
12	AtE301B01	at2g21380	kinesin motor proteinrelated	0.99
13	AtE301B02	at5g64920	COP1interacting protein (CIP8) / zinc finger (C3HC4type RING finger) family protein	1.30
14	AtE301B03	at3g55530	zinc finger (C3HC4type RING finger) family protein	2.70
15	AtE301B04	at2g39100	zinc finger (C3HC4type RING finger) family protein	3.13
16	AtE301B05	at2g39720	zinc finger (C3HC4type RING finger) family protein	3.31
17	AtE301B06	at1g74410	zinc finger (C3HC4type RING finger) family protein	2.07
18	AtE301B07	at5g07270	ankyrin repeat family protein	4.14
19	AtE301B08	at1g73760	zinc finger (C3HC4type RING finger) family protein	4.76
20	AtE301B10	at3g19910	zinc finger (C3HC4type RING finger) family protein	1.18
21	AtE301B11	at1g05890	zinc finger proteinrelated	3.19
22	AtE301B12	at1g80400	zinc finger (C3HC4type RING finger) family protein	2.81
23	AtE301C01	at1g15100	zinc finger (C3HC4type RING finger) family protein	5.32
24	AtE301C02	at5g19080	zinc finger (C3HC4type RING finger) family protein	1.47
25	AtE301C04	at3g54360	expressed protein	1.00
26	AtE301C05	at4g08460	expressed protein	4.04
27	AtE301C06	at2g18670	zinc finger (C3HC4type RING finger) family protein	3.46
28	AtE301C07	at1g72220	zinc finger (C3HC4type RING finger) family protein	2.88
29	AtE301C08	at1g71980	proteaseassociated zinc finger (C3HC4type RING finger) family protein	4.92
30	AtE301C09	at3g47550	zinc finger (C3HC4type RING finger) family protein	3.30
31	AtE301C10	at5g47050	expressed protein	1.72
32	AtE301C11	at2g47700	zinc finger (C3HC4type RING finger) family protein	4.68
33	AtE301C12	at1g08190	vacuolar assembly protein, putative (VPS41)	1.82
34	AtE301D01	at1g04790	zinc finger (C3HC4type RING finger) family protein	1.19
35	AtE301D02	at3g05670	PHD finger family protein	1.06
36	AtE301D03	at4g32600	zinc finger (C3HC4type RING finger) family protein	3.40
37	AtE301D04	at3g62240	zinc finger (C2H2 type) family protein	1.38
38	AtE301D05	at2g30580	zinc finger (C3HC4type RING finger) family protein	1.64
39	AtE301D06	at5g42940	zinc finger (C3HC4type RING finger) family protein	1.09
40	AtE301D07	at1g74760	zinc finger (C3HC4type RING finger) family protein	2.95
41	AtE301D08	at5g10380	zinc finger (C3HC4type RING finger) family protein	4.72
42	AtE301D09	at3g23280	zinc finger (C3HC4type RING finger) family protein / ankyrin repeat family protein	1.81
43	AtE301D10	at5g22920	zinc finger (C3HC4type RING finger) family protein	1.23
44	AtE301D11	at5g41350	zinc finger (C3HC4type RING finger) family protein	1.01
45	AtE301D12	at5g56340	zinc finger (C3HC4type RING finger) family protein	1.50
46	AtE301E01	at2g04240	zinc finger (C3HC4type RING finger) family protein	3.93
47	AtE301E02	at3g05200	zinc finger (C3HC4type RING finger) family protein (ATL6)	1.96
48	AtE301E03	at2g44410	expressed protein	3.25
49	AtE301E04	at2g45530	zinc finger (C3HC4type RING finger) family protein	2.19
50	AtE301E05	at4g39140	expressed protein	2.07
51	AtE301E06	at4g19700	expressed protein	1.60
52	AtE301E07	at3g60300	RWD domaincontaining protein	3.31
53	AtE301E08	at2g32950	COP1 regulatory protein	3.70
54	AtE301E09	at2g44950	zinc finger (C3HC4type RING finger) family protein	2.66
55	AtE301E10	at4g35480	zinc finger (C3HC4type RING finger) family protein	0.86
56	AtE301E11	at5g45100	expressed protein	1.37
57	AtE301E12	at5g45290	zinc finger (C3HC4type RING finger) family protein	2.11
58	AtE301F01	at5g19430	zinc finger (C3HC4type RING finger) family protein	2.51
59	AtE301F02	at5g60580	zinc finger (C3HC4type RING finger) family protein	1.81
60	AtE301F03	at1g79380	copinerelated	0.64
61	AtE301F04	at5g66160	proteaseassociated zinc finger (C3HC4type RING finger) family protein	2.59
62	AtE301F05	at5g01520	zinc finger (C3HC4type RING finger) family protein	4.09
63	AtE301F06	at2g15580	zinc finger (C3HC4type RING finger) family protein	0.97
64	AtE301F07	at4g30400	zinc finger (C3HC4type RING finger) family protein	2.76
65	AtE301F08	at5g03180	zinc finger (C3HC4type RING finger) family protein	1.92
66	AtE301F09	at1g06770	zinc finger (C3HC4type RING finger) family protein	4.27
67	AtE301F10	at2g05170	vacuolar protein sorting 11 family protein / VP1 family protein	2.24
68	AtE301F11	at1g32530	zinc finger (C3HC4type RING finger) family protein	2.45
69	AtE301F12	at5g63970	copinerelated	2.84
70	AtE301G01	at1g21650	preprotein translocase secA family protein	3.27
71	AtE301G02	at5g18650	zinc finger (C3HC4type RING finger) family protein	3.24
72	AtE301G03	at1g79810	Pex2/Pex12 Nterminal domaincontaining protein / zinc finger (C3HC4type RING finger) family protein	3.50
73	AtE301G04	at2g21500	expressed protein	2.00
74	AtE301G05	at2g35330	zinc finger (C3HC4type RING finger) proteinrelated	1.26
75	AtE301G06	at2g40830	zinc finger (C3HC4type RING finger) family protein	9.46
76	AtE301G07	at3g46620	zinc finger (C3HC4type RING finger) family protein	3.14
77	AtE301G08	at1g54150	zinc finger (C3HC4type RING finger) family protein	1.72
78	AtE301G09	at1g63170	zinc finger (C3HC4type RING finger) family protein	1.05
79	AtE301G10	at2g37150	zinc finger (C3HC4type RING finger) family protein	1.61
80	AtE301G11	at1g57820	zinc finger (C3HC4type RING finger) family protein	2.62
81	AtE301G12	at3g61460	zinc finger (C3HC4type RING finger) family protein (BRH1)	4.02
82	AtE301H01	at3g09770	zinc finger (C3HC4type RING finger) family protein	2.65
83	AtE301H02	at4g25230	zinc finger (C3HC4type RING finger) family protein	4.60
84	AtE301H03	at3g10810	zinc finger (C3HC4type RING finger) family protein	3.85
85	AtE301H04	at1g12760	zinc finger (C3HC4type RING finger) family protein	1.52
86	AtE301H05	at5g27420	zinc finger (C3HC4type RING finger) family protein	3.40
87	AtE301H06	at1g26800	zinc finger (C3HC4type RING finger) family protein	2.29
88	AtE301H07	at2g31510	IBR domaincontaining protein / ARIADNElike protein ARI7 (ARI7)	1.91
89	AtE301H08	at5g06420	zinc finger (CCCHtype/C3HC4type RING finger) family protein	1.31
90	AtE301H09	at5g15820	zinc finger (C3HC4type RING finger) family protein	2.18
91	AtE301H10	at4g23450	zinc finger (C3HC4type RING finger) family protein	1.07
92	AtE301H11	at2g02960	zinc finger (C3HC4type RING finger) family protein	2.92
93	AtE301H12	at1g55530	zinc finger (C3HC4type RING finger) family protein	0.98
94	AtE302A01	at2g28840	ankyrin repeat family protein	5.61
95	AtE302A02	at4g39050	kinesinrelated protein (MKRP2)	1.89
96	AtE302A03	at3g47160	expressed protein	3.28
97	AtE302A04	at5g20910	zinc finger (C3HC4type RING finger) family protein	1.57
98	AtE302A05	at5g14420	copinerelated	2.16
99	AtE302A06	at3g18930	zinc finger (C3HC4type RING finger) family protein	3.31
100	AtE302A07	at5g10650	zinc finger (C3HC4type RING finger) family protein	3.33
101	AtE302A08	at4g11680	zinc finger (C3HC4type RING finger) family protein	2.29
102	AtE302A09	at1g19310	zinc finger (C3HC4type RING finger) family protein	3.77
103	AtE302A10	at5g51450	zinc finger (C3HC4type RING finger) family protein	4.13

104	AtE302A11	at1g17970	zinc finger (C3HC4type RING finger) family protein	2.25
105	AtE302A12	at3g61550	zinc finger (C3HC4type RING finger) family protein	3.96
106	AtE302B01	at3g06330	zinc finger (C3HC4type RING finger) family protein	1.24
107	AtE302B02	at3g27710	zinc finger proteinrelated	1.32
108	AtE302B03	at3g26730	zinc finger (C3HC4type RING finger) family protein	1.70
109	AtE302B04	at3g16720	zinc finger (C3HC4type RING finger) family protein	1.94
110	AtE302B05	at5g18260	expressed protein	2.29
111	AtE302B06	at5g01960	zinc finger (C3HC4type RING finger) family protein	1.93
112	AtE302B07	at5g37890	seven in absentia (SINA) protein, putative	1.97
113	AtE302B08	at3g18290	zinc finger proteinrelated	2.53
114	AtE302B09	at3g54460	SNF2 domaincontaining protein / helicase domaincontaining protein / Fbox family protein	2.53
115	AtE302B10	at5g62460	zinc finger (C3HC4type RING finger) family protein	3.26
116	AtE302B11	at1g69330	zinc finger (C3HC4type RING finger) family protein	4.60
117	AtE302B12	at2g35910	zinc finger (C3HC4type RING finger) family protein	4.69
118	AtE302C01	at2g22680	zinc finger (C3HC4type RING finger) family protein	1.77
119	AtE302C02	at2g42030	zinc finger (C3HC4type RING finger) family protein	2.38
120	AtE302C03	at4g26400	zinc finger (C3HC4type RING finger) family protein	3.83
121	AtE302C04	at5g63780	zinc finger (C3HC4type RING finger) family protein	3.93
122	AtE302C05	at5g63760	IBR domaincontaining protein	3.82
123	AtE302C06	at3g23060	zinc finger (C3HC4type RING finger) family protein	4.40
124	AtE302C07	at3g61180	zinc finger (C3HC4type RING finger) family protein	3.17
125	AtE302C08	at5g01160	ecadherin binding proteinrelated	3.33
126	AtE302C09	at3g58030	zinc finger (C3HC4type RING finger) family protein	4.44
127	AtE302C10	at1g18660	zinc finger (C3HC4type RING finger) family protein	3.54
128	AtE302C11	at5g60710	zinc finger (C3HC4type RING finger) family protein	1.94
129	AtE302C12	at1g19680	expressed protein	2.16
130	AtE302D01	at3g02340	zinc finger (C3HC4type RING finger) family protein	2.77
131	AtE302D02	at1g59560	expressed protein	5.08
132	AtE302D03	at2g42360	zinc finger (C3HC4type RING finger) family protein	5.23
133	AtE302D04	at5g57740	zinc finger (C3HC4type RING finger) family protein / ankyrin repeat family protein	3.52
134	AtE302D05	at1g08050	zinc finger (C3HC4type RING finger) family protein	2.27
135	AtE302D06	at3g29270	expressed protein	2.07
136	AtE302D07	at5g42200	zinc finger (C3HC4type RING finger) family protein	1.14
137	AtE302D08	at1g77770	expressed protein	1.13
138	AtE302D09	at1g74370	zinc finger (C3HC4type RING finger) family protein	1.14
139	AtE302D10	at5g37930	seven in absentia (SINA) family protein	1.97
140	AtE302D11	at2g47560	zinc finger (C3HC4type RING finger) family protein	2.83
141	AtE302D12	at1g72200	zinc finger (C3HC4type RING finger) family protein	3.43
142	AtE302E01	at2g22120	zinc finger (C3HC4type RING finger) family protein	5.30
143	AtE302E02	at3g61790	seven in absentia (SINA) family protein	3.99
144	AtE302E03	at1g18470	zinc finger (C3HC4type RING finger) family protein	3.56
145	AtE302E04	at5g22750	SNF2 domaincontaining protein / helicase domaincontaining protein / RING finger domaincontaining protein	6.03
146	AtE302E05	at1g47570	zinc finger (C3HC4type RING finger) family protein	7.84
147	AtE302E06	at2g35000	zinc finger (C3HC4type RING finger) family protein	4.69
148	AtE302E07	at4g19670	zinc finger (C3HC4type RING finger) family protein	1.54
149	AtE302E08	at5g44280	zinc finger (C3HC4type RING finger) family protein	2.55
150	AtE302E09	at4g09560	proteaseassociated zinc finger (C3HC4type RING finger) family protein	4.16
151	AtE302E10	at5g59000	zinc finger (C3HC4type RING finger) family protein	4.99
152	AtE302E12	at4g03000	expressed protein	5.14
153	AtE302F01	at5g15790	zinc finger (C3HC4type RING finger) family protein	3.85
154	AtE302F02	at5g01880	zinc finger (C3HC4type RING finger) family protein	3.45
155	AtE302F03	at4g34040	zinc finger (C3HC4type RING finger) family protein	4.15
156	AtE302F04	at1g60610	expressed protein	4.36
157	AtE302F05	at3g60080	zinc finger (C3HC4type RING finger) family protein	2.86
158	AtE302F06	at3g45630	RNA recognition motif (RRM)containing protein	2.51
159	AtE302F07	at3g16090	zinc finger (C3HC4type RING finger) family protein	2.55
160	AtE302F08	at1g61620	expressed protein	2.21
161	AtE302F09	at5g03200	zinc finger (C3HC4type RING finger) family protein	1.45
162	AtE302F10	at3g58040	seven in absentia (SINA) family protein	1.23
163	AtE302F11	at4g37890	zinc finger (C3HC4type RING finger) family protein	1.37
164	AtE302F12	at3g47990	zinc finger (C3HC4type RING finger) family protein	3.22
165	AtE302G01	at1g11020	zinc finger (C3HC4type RING finger) family protein	4.34
166	AtE302G02	at5g53360	seven in absentia (SINA) family protein	2.62
167	AtE302G03	at5g57820	hypothetical protein	1.32
168	AtE302G04	at1g75400	expressed protein	1.54
169	AtE302G05	at1g50440	zinc finger (C3HC4type RING finger) family protein	1.85
170	AtE302G06	at3g05250	zinc finger (C3HC4type RING finger) family protein	2.29
171	AtE302G07	at2g26350	zincbinding peroxisomal integral membrane protein (PEX10)	3.22
172	AtE302G08	at4g14220	zinc finger (C3HC4type RING finger) family protein	4.72
173	AtE302G10	at1g32360	zinc finger (CCCHtype) family protein	4.47
174	AtE302G11	at4g35840	zinc finger (C3HC4type RING finger) family protein	2.71
175	AtE302G12	at5g20570	ringbox proteinrelated	3.85
176	AtE302H02	at5g02750	zinc finger (C3HC4type RING finger) family protein	3.78
177	AtE302H03	at3g48030	hypoxiaresponsive family protein / zinc finger (C3HC4type RING finger) family protein	3.43
178	AtE302H04	at4g22250	zinc finger (C3HC4type RING finger) family protein	4.51
179	AtE302H05	at3g07200	zinc finger (C3HC4type RING finger) family protein	6.13
180	AtE302H06	at3g12920	expressed protein	4.43
181	AtE302H07	at2g27940	zinc finger (C3HC4type RING finger) family protein	2.89
182	AtE302H08	at2g17730	zinc finger (C3HC4type RING finger) family protein	2.19
183	AtE302H09	at5g18760	zinc finger (C3HC4type RING finger) family protein	1.55
184	AtE302H10	at1g76410	zinc finger (C3HC4type RING finger) family protein	2.25
185	AtE302H11	at4g30370	zinc finger (C3HC4type RING finger) family protein	2.18
186	AtE302H12	at5g05530	zinc finger (C3HC4type RING finger) family protein	2.79
187	AtE303A01	at1g23980	zinc finger (C3HC4type RING finger) family protein	5.11
188	AtE303A02	at4g33940	zinc finger (C3HC4type RING finger) family protein	4.44
189	AtE303A03	at2g38920	SPX (SYG1/Pho81/XPR1) domaincontaining protein / zinc finger (C3HC4type RING finger) proteinrelated	3.36
190	AtE303A04	at1g74870	expressed protein	0.80
191	AtE303A05	at3g48070	expressed protein	1.74
192	AtE303A06	at3g43430	zinc finger (C3HC4type RING finger) family protein	4.27
193	AtE303A07	at4g08590	zinc finger (C3HC4type RING finger) family protein	1.67
194	AtE303A08	at3g30460	zinc finger (C3HC4type RING finger) family protein	1.15
195	AtE303A09	at1g66050	zinc finger (C3HC4type RING finger) family protein	4.47
196	AtE303A10	at1g79110	expressed protein	2.42
197	AtE303A11	at1g30860	expressed protein	2.18
198	AtE303A12	at5g40250	zinc finger (C3HC4type RING finger) family protein	3.26
199	AtE303B01	at5g01980	zinc finger (C3HC4type RING finger) family protein	2.85
200	AtE303B02	at1g49230	zinc finger (C3HC4type RING finger) family protein	1.21
201	AtE303B03	at1g05880	expressed protein	3.53
202	AtE303B04	at5g47610	zinc finger (C3HC4type RING finger) family protein	3.15
203	AtE303B05	at5g39550	zinc finger (C3HC4type RING finger) family protein	3.76
204	AtE303B06	AT3g25030	RING/Ubox superfamily protein	1.00

All data are the average of two independent experiments, and the background was controlled for biotinylated DHFR.

Supplementary Table 2: List of primers for plasmid construct

AGI code	gene name	primer name	primer sequence (5'-3')	Remarks
**Cloning**				
AT3G05120	GID1A	S1-GID1A	CCACCCACCACCACCAATGGCTGCGAGCGATGAAGT	underline indicate S1-linker sequence
		T1-GID1A	TCCAGCACTAGCTCCAGATTAACATTCCGCGTTTAC	underline indicate T1-linker sequence
		T1-GID1A w/o stop	TCCAGCACTAGCTCCAGAACATTCCGCGTTTACAAACG	underline indicate T1-linker sequence
AT3G63010	GID1B	S1-GID1B	CCACCCACCACCACCAATGGCTGGTGGTAACGAAGT	underline indicate S1-linker sequence
		T1-GID1B	TCCAGCACTAGCTCCAGACTAAGGAGTAAGAAGCACAG	underline indicate T1-linker sequence
AT5G27320	GID1C	S1-GID1C	CCACCCACCACCACCAATGGCTGGAAGTGAAGAAGT	underline indicate S1-linker sequence
		T1-GID1C	TCCAGCACTAGCTCCAGATCATTGGCATTCTGCGTTTAC	underline indicate T1-linker sequence
AT2G40830	GARU	S1-GARU	CCACCCACCACCACCAATGTCAAGCAGTCGAAATAC	underline indicate S1-linker sequence
		T1-GARU	TCCAGCACTAGCTCCAGATTAGTAATCAAAAGGCCAGC	underline indicate T1-linker sequence
AT4G17870	PYR1	S1-PYR1	CCACCCACCACCACCAATGCCTTCGGAGTTAACACC	underline indicate S1-linker sequence
		T1-PYR1	TCCAGCACTAGCTCCAGATCACGTCACCTGAGAACCAC	underline indicate T1-linker sequence
AT5G46790	PYL1	S1-PYL1	CCACCCACCACCACCAATGCCTTCCGATTTAACTCA	underline indicate S1-linker sequence
		T1-PYL1	TCCAGCACTAGCTCCAGATTACCTAACCTGAGAAGAGT	underline indicate T1-linker sequence
AT1G14920	GAI	S1-GAI	CCACCCACCACCACCAATGAAGAGAGATCATCATCA	underline indicate S1-linker sequence
		T1-GAI	TCCAGCACTAGCTCCAGACTAATTGGTGGAGAGTTTCC	underline indicate T1-linker sequence
AT3G03450	RGL2	S1-RGL2	CCACCCACCACCACCAATGAAGAGAGGATACGGAGA	underline indicate S1-linker sequence
		T1-RGL2	TCCAGCACTAGCTCCAGATCAGGCGAGTTTCCACGCCG	underline indicate T1-linker sequence
AT4G05320	UBQ10	S1-UBQ10	CCACCCACCACCACCAATGCAGATCTTCGTTAAGAC	underline indicate S1-linker sequence
		T1-UBQ10	TCCAGCACTAGCTCCAGATTAACCACCACGGAGCCTGAG	underline indicate T1-linker sequence
		attB1-S1	GGGGACAAGTTTGTACAAAAAAGCAGGCTTCCACCCACCACCACCAATG	underline indicate S1-linker sequence
		attB2-T1	GGGGACCACTTTGTACAAGAAAGCTGGGTCTCCAGCACTAGCTCCAGA	underline indicate T1-linker sequence
		attB2-STOP-His-T1	GGGGACCACTTTGTACAAGAAAGCTGGGTTTAATGATGGTGATGGTGATG GCTTCCAGCACTAGCTCCAGA	underline indicate His-tag epitope sequence
		attB2-STOP-AGIA-T1	GGGGACCACTTTGTACAAGAAAGCTGGGTTTACAATGGGCGAGCAATACC AGCTGCTTCTTCTCCAGCACTAGCTCCAGA	underline indicate AGIA-tag epitope sequence
		S1-HA-UBQ10	CCACCCACCACCACCAATGATGACTAGC**TACCCTTATGACGTACCCGATTA CGCC**ATGCAGATCTTTGTTAAGA	bold indicate indicateHA-tag epitope sequence
		R-UBQ-SpeI	TAAGGGTAACTAGTCATACCACCACGGAGCCTGAGG	underline indicate SpeI site
		F-UBQ-SpeI	ATGACTAGT**TACCCTTATGACGTACCCGATTACGCC**	bold indicate indicateHA-tag epitope sequence, underline indicate SpeI site
		R-UBQ-AatII	**GGCGTAATCGGGGACGTCATAAGGGTA**GCTAGTCATACCACCACGGAGC CTGAG	bold indicate indicateHA-tag epitope sequence, underline indicate AatII site
		F-UBQ-AatII	ATGACTAGC**TACCCTTATGACGTCCCCGATTACGCC**	bold indicate indicateHA-tag epitope sequence, underline indicate AatII site
		T1-UBQ10	TCCAGCACTAGCTCCAGATTAACCACCACGGAGCCTGAG	
**Vector constluct**				
		F-pEU-V5-InFusion	GGTAAGCCTATCCCTAACCCTCTCCTCGGTCTCGATTCTACGTAGCTAGCT AGATATCACTAG	underline indicate V5-tag epitope sequence
		R-pEU-V5-InFusion	CGTAGAATCGAGACCGAGGAGAGGGTTAGGGATAGGCTTACCCACCACTT TGTACAAGAAAGC	underline indicate V5-tag epitope sequence
		F-pEU-HA-InFusion	TACCCATACGATGTTCCAGATTACGCTACAAGTTTGTACAAAAAAGC	underline indicate HA-tag epitope sequence
		R-pEU-HA-InFusion	AGCGTAATCTGGAACATCGTATGGGTACATGATATCTTGGTGATGTAG	underline indicate HA-tag epitope sequence
		F-pEU-bls-InFusion	AACGACATCTTCGAGGCCCAGAAGATCGAGTGGCACGAAACAAGTTTGTA CAAAAAAGC	underline indicate bls-tag epitope sequence
		R-pEU-bls-InFusion	CTCGAAGATGTCGTTCAGGCC**ATGATGGTGATGGTGATG**CATGATATCTTG GTGATGTAG	underline indicate bls-tag epitope sequence, bold indicate His tag sequence
		F-pEU-cassettes	CTAGTTAAGCTATCATACATCACCAAGATATCATG	GW-cassettes
		R-pEU-cassettes	TCTAAGTAGTTGATTGCTCGAGAACTAGTGATATC	GW-cassettes
		F-35SΩ linialized	TGATCTAGAGGGCCCGCGGTTCGAA	p35SΩ-GW-NOST linialized
		R-35SΩ linialized	TGATAGCTTAACTAGCCAGCTTGGG	p35SΩ-GW-NOST linialized
		F-3FLAG-ClaI	ACGAC**ATCGATTACAAGGATGACGATGACAAG**TAAAGCGGCCGCCCGGG C	underline indicate ClaI site, bold indicate 3× FLAG-tag epitope sequence
		R-3FLAG-ClaI	TTGTA**ATCGATGTCGTGATCTTTATAGTCCCCATCGTGATCCTTGTAGTC**TT CGAACCGCGGGCCCTCTAG	underline indicate ClaI site, bold indicate 3× FLAG-tag epitope sequence
		F-YFP	ATGGTGAGCAAGGGCGAGGAG	YFP
		R-YFP	CTTGTACAGCTCGTCCATGCCG	YFP
		F-35SΩ-V5 linialized	GACGAGCTGTACAAGGGTAAGCCTATCCCTAACCC	p35SΩ-V5-GW-NOST linialized
		R-35SΩ-V5 linialized	GCCCTTGCTCACCATGATATCTTGGTGATGTATCC	p35SΩ-V5-GW-NOST linialized
**Mutation**				
		F-GARU-C190-193S	CTCGAATAGTCCCGTGaGcAAAGACGAATTCGAACTGGG	C190S and C193S
		R-GARU-C190-193S	GTCTTTGCtCACGGGACtATTCGAGTCCGATGACCTAAG	C190S and C193S
		F-GARU-del-Cter	AACAATCATAAGACCCAGCTTTCTTG	C-terminal deletion
		R-GARU-del-Cter	GGTCTTATGATTGTTGATGTTGCTG	C-terminal deletion
		F-GARU-del-Nter	CAGTCGAGATATGGCTCAAGCCAGCC	N-terminal deletion
		R-GARU-del-Nter	CCATATCTCGACTGCTTGACAT	N-terminal deletion
		F-GARU-Y28F	TGCTTTTTCTGCGGAGGTGGATTTG	Y28F

	R-GARU-Y28F	TCCGCAGAAAAAGCATACAGGCTC	Y28F
	F-GARU-Y140F	GGCGACTTCTTCTTCGGTCCCGGC	Y140F
	R-GARU-Y140F	GAAGAAGAAGTCGCCGGTGTTACC	Y140F
	F-GARU-Y212F	CACATCTTCCATTCTGACTGCATT	Y212F
	R-GARU-Y212F	AGAATGGAAGATGTGGTTACACGG	Y212F
	F-GARU-Y253F	AGAAACTTCAGAAGCAGTAGTAGT	Y253F
	R-GARU-Y253F	GCTTCTGAAGTTTCTGGTAGGGGT	Y253F
	F-GARU-Y309F	CATAACTTCCATCAACAGCAACAT	Y309F
	R-GARU-Y309F	TTGATGGAAGTTATGGTTCTCATC	Y309F
	F-GARU-Y321F	ATGGGTTTCAGTGGCTGGCCTTTTG	Y321F
	R-GARU-Y321F	GCCACTGAAACCCATATATGATTG	Y321F
	F-GARU-Y328F	TTTGATTTCTAATCTGGAGCTAGT	Y328F
	R-GARU-Y328F	AGATTAGAAATCAAAAGGCCAGCC	Y328F
	F-GARU-Y321D	ATGGGTGATAGTGGCTGGCCTTTTG	Y321D
	R-GARU-Y321D	GCCACTATCACCCATATATGATTGTTG	Y321D
	F-GARU-Y321E	ATGGGTGAGAGTGGCTGGCCTTTTG	Y321E
	R-GARU-Y321E	GCCACTCTCACCCATATATGATTGTTG	Y321E
	F-TAGK3-M224A	ATAGTCGCAGAGTTGTGTGATGGTGGA	M224A
	R-TAGK3-M224A	CAACTCTGCGACTATGTATACGTTATT	M224A
	F-TAGK3-C227A	GAGTTGGCAGATGGTGGAGAACTTTTG	C227A
	R-TAGK3-C227A	ACCATCTGCCAACTCCATGACTATGTA	C227A
**RT-qPCR**			
AT3G05120	F-GID1a	GCTGCGAGCGATGAAGTTA	
	R-GID1a	AACCCATGTATTGAGAGGAACC	
AT3G63010	F-GID1b	GCTGCGAGCGATGAAGTTA	
	R-GID1b	AACCCATGTATTGAGAGGAACC	
AT5G27320	F-GID1c	GCTGCGAGCGATGAAGTTA	
	R-GID1c	AACCCATGTATTGAGAGGAACC	
AT2G40830	F-GARU	GACGGAGAGATTCTTCTAGAGTCAG	
	R-GARU	TTTGACAGGTCTCAGTTAAAAACCT	
AT5G25760	F-UBC21	CCGCTCTTTCTTTCCAAGC	
	R-UBC21	CCGGTACCATTGTCACACAC	
AT3G18780	F-ACTIN2	CCGCTCTTTCTTTCCAAGC	
	R-ACTIN2	CCGGTACCATTGTCACACAC	

Supplementary Table 3: List of plasmids for cell-free expression, transient expression and Agrobacterium-mediated transformation

Plasmid name	Expressed Protein
**Cell-free expression plasmids**	
pEU-E01-GID1A-His	GID1A-His
pEU-E01-GID1A-AGIA	GID1A-AGIA
pEU-E01-GID1A K0-His	GID1A^{K0}-His
pEU-E01-GID1A K0-AGIA	GID1A^{K0}-AGIA
pEU-E01-V5-GID1A	V5-GID1A
pEU-E01-V5-GID1B	V5-GID1B
pEU-E01-V5-GID1C	V5-GID1C
pEU-E01-His-bls-GID1A	biotin-GID1A
pEU-E01-V5-PYL1	V5-PYL1
pEU-E01-GID1A-V5	GID1A-V5
pEU-E01-FLAG-GARU	FLAG-GARU
pEU-E01-FLAG-GARU CS	FLAG-GARU^{CS}
pEU-E01-FLAG-GARU ΔN	FLAG-GARU ΔN
pEU-E01-FLAG-GARU ΔC	FLAG-GARU ΔC
pEU-E01-FLAG-GARU Y28F	FLAG-GARU^{Y28F}
pEU-E01-FLAG-GARU Y140F	FLAG-GARU^{Y140F}
pEU-E01-FLAG-GARU Y212F	FLAG-GARU^{Y212F}
pEU-E01-FLAG-GARU Y253F	FLAG-GARU ^{Y253F}
pEU-E01-FLAG-GARU Y309F	FLAG-GARU^{Y309F}
pEU-E01-FLAG-GARU Y321F	FLAG-GARU ^{Y321F}
pEU-E01-FLAG-GARU Y328F	FLAG-GARU ^{Y328F}
pEU-E01-FLAG-GARU Y321D	FLAG-GARU ^{Y321D}
pEU-E01-FLAG-GARU Y321E	FLAG-GARU ^{Y321E}
pEU-E01-V5-GARU	V5-GARU
pEU-E01-His-bls-TAGK2	biotin-TAGK2
pEU-E01-His-bls-TAGK3	biotin-TAGK3
pEU-E01-His-bls-GmTAGK3	biotin-GmTAGK3
pEU-E01-His-bls-ERF13	biotin-ERF13
pEU-E01-His-bls-TAGK3 M224A	biotin-TAGK3 M224A
pEU-E01-His-bls-TAGK3 C227A	biotin-TAGK3 C227A
pEU-E01-FLAG-TAGK2	FLAG-TAGK2
pEU-E01-FLAG-TAGK3	FLAG-TAGK3
pEU-E01-FLAG-TAGK2 KD	FLAG-TAGK2 ^{KD}
pEU-E01-FLAG-TAGK3 KD	FLAG-TAGK3 ^{KD}
pEU-E01-V5-PYR1	V5-PYR1
pEU-E01-His-bls-GAI	biotin-GAI
pEU-E01-FLAG-GAI	FLAG-GAI
pEU-E01-His-bls-RGL2	biotin-RGL2
pEU-E01-His-bls-DHFR	biotin-DHFR
pEU-E01-GW	Gateway destination expression vector[1]
pEU-E01-V5-GW	Gateway destination expression vector (in this study)
pEU-E01-HA-GW	Gateway destination expression vector (in this study)
pEU-E01-His-bls-GW	Gateway destination expression vector (in this study)
pEU-E01-FLAG-GW	Gateway destination expression vector[1]
pEU-E01-AGIA-GW	Gateway destination expression vector[1]
**Transient expression plasmids**	
p35SΩ-TAGK2-His-NOST	TAGK2-His
p35SΩ-TAGK2KD-His-NOST	TAGK2^{KD}-His
p35SΩ-GID1A-AGIA-NOST	GID1A-AGIA
p35SΩ-3× (HA-UBQ10)-NOST	3× (HA-UBQ10)
p35SΩ-V5-GARU-NOST	V5-GARU
p35SΩ-V5-GARU CS-NOST	V5-GARU^{CS}
p35SΩ-AGIA-GARU CS-NOST	AGIA-GARU^{CS}
p35SΩ-AGIA-GARU CS Y321F-NOST	AGIA-GARU $^{CS/Y321F}$
p35SΩ-V5-GARU Y321E-NOST	V5-GARU ^{Y321E}
p35SΩ-V5-GARU Y321F-NOST	V5-GARU ^{Y321F}
p35SΩ-V5-GARU Y321D-NOST	V5-GARU ^{Y321D}
p35SΩ-TAGK2-HA-NOST	TAGK2-HA
p35SΩ-TAGK3-HA-NOST	TAGK3-HA
p35SΩ-TAGK2 KD-HA-NOST	TAGK2 ^{KD}-HA
p35SΩ-TAGK3 KD-HA-NOST	TAGK3 ^{KD}-HA
p35SΩ-FLAG-ERF13-NOST	FLAG-ERF13
p35SΩ-TAGK2-3× FLAG-NOST	TAGK2-3× FLAG
p35SΩ-TAGK2KD-3× FLAG-NOST	TAGK2^{KD}-3× FLAG
p35SΩ-YFP-V5-GARU-NOST	YFP-V5-GARU
p35SΩ-GID1A-GFP-NOST	GID1A-GFP
p35SΩ-GID1A-cYFP-NOST	GID1A-cYFP
p35SΩ-nYFP-GARU-NOST	nYFP-GARU
p35SΩ-cYFP-NOST	cYFP
p35SΩ-nYFP-NOST	nYFP
p35SΩ-GW-NOST	Gateway destination expression vector[2]
p35SΩ-GW-GFP-NOST	Gateway destination expression vector[2]
p35SΩ-V5-GW-NOST	Gateway destination expression vector (in this study)
p35SΩ-AGIA-GW-NOST	Gateway destination expression vector (in this study)
p35SΩ-GW-3×FALG-NOST	Gateway destination expression vector (in this study)
p35SΩ-YFP-V5-GW-NOST	Gateway destination expression vector (in this study)
**Agrobacterium-mediated transformation**	
pBCR-35S:::TAGK2^{WT}-HA	TAGK2^{WT}-HA
pBCR-35S:::TAGK2^{KD}-HA	TAGK2^{KD}-HA

Supplementary Table 4: List of the antibodies used in this study

antibody or beads name	souce	Cat. NO.	Diluted
Rabbit polyclonal anti-RGA	Agrisera	AS11 1630	1/1000
Rabbit polyclonal anti-GID1A	In this study	N/A	1/1000
Rabbit monoclonal anti-AGIA	Previous our study[1]	N/A	1/10000
Rabbit monoclonal anti-AGIA, HRP conjugated	Previous our study[1]	N/A	1/10000
Mouse monoclonal anti-phosphotyrosine (4G10), HRP conjugated	Millipore	16-105	1/5000
Mouse monoclonal anti-FLAG M2	Sigma-Aldrich	F3165	1/5000
Mouse monoclonal anti-FLAG M2, HRP conjugated	Sigma-Aldrich	A8592	1/5000
Mouse monoclonal anti-V5, HRP conjugate	Thermo Fisher Scientific	R961-25	1/10000
Rat monoclonal anti-HA (3F10), HRP conjugated	Roche Applied Science	12013819001	1/5000
Streptavidin, Alexa Fluor 647 conjugated	Thermo Fisher Scientific	S-21374	1/5000
Mouse monoclonal anti-Biotin, HRP conjugated	Sigma-Aldrich	A0185	1/10000
Mouse monoclonal anti-His	GE Healthcare	27-4710-01	1/1000
Mouse monoclonal anti-ubiquitin (P4D1)	Santa Cruz Biotechnology	sc-8017	1/1000
Mouse monoclonal anti-αtubulin (DM1A)	Abcam	ab7291	1/10000
Sheep Anti-Mouse IgG ECL Antibody, HRP Conjugated	GE Healthcare	NA9310-1ML	1/10000
Donkey Anti-Rabbit IgG ECL Antibody, HRP Conjugated	GE Healthcare	NA9340-1ML	1/10000
Anti-FLAG M2 affinity agarose gel	Sigma-Aldrich	A2220	none
Anti-AGIA antibody sepharose beads	Previous our study[1]	N/A	none
Dynabeads M-280 streptavidin	Thermo Fisher Scientific	11205D	none

Supplementary References

1. Yano, T. *et al.* AGIA Tag System Based on a High Affinity Rabbit Monoclonal Antibody against Human Dopamine Receptor D1 for Protein Analysis. *PLoS One* **11**, e0156716 (2016).

2. Nemoto, K., Takemori, N., Seki, M., Shinozaki, K. & Sawasaki, T. Members of the Plant CRK Superfamily Are Capable of Trans- and Autophosphorylation of Tyrosine Residues. *J. Biol. Chem.* **290**, 16665-16677 (2015).
